# Local delivery of soluble fractalkine (CX3CL1) peptide restores ribbon synapses after noise-induced cochlear synaptopathy

**DOI:** 10.3389/fncel.2024.1486740

**Published:** 2024-10-30

**Authors:** Vijayprakash Manickam, Sibaprasad Maity, Sree Varshini Murali, Dinesh Y. Gawande, Andrew R. Stothert, Lyudamila Batalkina, Astrid E. Cardona, Tejbeer Kaur

**Affiliations:** ^1^Department of Biomedical Sciences, Creighton University, Omaha, NE, United States; ^2^Department of Otolaryngology, Robert Wood Johnson Medical School, Rutgers University, Piscataway, NJ, United States; ^3^Department of Molecular Microbiology and Immunology, University of Texas, San Antonio, TX, United States

**Keywords:** cochlea, ribbon synapses, hidden hearing loss, noise trauma, fractalkine, macrophages

## Abstract

Cochlear ribbon synapses between sensory inner hair cells (IHCs) and spiral ganglion neurons (SGNs) are vulnerable to rapid and primary damage and/or loss due to noise overexposure. Such damaged ribbon synapses can repair spontaneously in mouse and guinea pig. However, the mechanisms for synaptic repair are unclear. Previously, we have demonstrated a critical role for the fractalkine signaling axis (CX_3_CL1-CX_3_CR1) in synaptic repair, wherein noise-damaged ribbon synapses are spontaneously repaired in the presence of fractalkine receptor (CX_3_CR1) expressed by cochlear macrophages. Here, we examined whether local administration of chemokine fractalkine ligand (CX_3_CL1 or FKN) in the form of a peptide is effective in restoring synapses and hearing loss after noise-induced cochlear synaptopathy (NICS). Specifically, the efficacy of different isoforms of FKN was evaluated for restoration of loss of IHC ribbon synapses and hearing after NICS. A single transtympanic injection of soluble isoform of FKN (sFKN) peptide at 1 day after synaptopathic noise trauma for 2 hours at 93 decibel sound pressure level led to significant recovery of auditory brainstem response (ABR) thresholds, ABR peak I amplitudes and ribbon synapses in FKN knockout mice when compared to mice injected with membrane-bound FKN peptide (mFKN). Likewise, local treatment with sFKN peptide in FKN wild type mice restored synaptopathic noise-damaged ribbon synapses and ABR peak I amplitudes. Mechanistically, FKN regulates macrophage numbers in the damaged cochlea and in the absence of macrophages, sFKN failed to restore loss of synapses and hearing after NICS. Furthermore, sFKN treatment attenuated cochlear inflammation after NICS without altering the expression of CX_3_CR1. Finally, injected sFKN peptide was detectable inside the cochlea for 24 h localized to the basilar membrane and spiral lamina near the sensory epithelium. These data provide a proof-of-principle that local delivery of an immune factor, sFKN is effective in restoring ribbon synapses and hearing loss after NICS in a macrophage-dependent manner and highlights the potential of sFKN as an immunotherapy for cochlear synaptopathy due to noise.

## Background

Nearly 30 million Americans (18%) aged 20–69 have hearing loss in both ears, and 48 million have hearing loss in at least one ear from exposure to loud noise (National Institute on Deafness and Other Communication Disorders, NIDCD). The prevalence of noise-induced hearing loss in the military population is more significant than in the general public. As per the Department of Veterans Affairs, there are an estimated 1.3 million Veterans with a Service-related disability due to hearing loss (Theodoroff et al., 2015). Noise exposure causes hearing loss due to damage to the sensory hair cells of the inner ear (Webster and Webster, 1981) or due to loss of synaptic contacts (a.k.a. ribbon synapse) between inner hair cells (IHCs) and peripheral dendrites of the spiral ganglion neurons (SGNs) (Puel et al., 1998; Kujawa and Liberman, 2009). Synaptic loss can trigger gradual degeneration of peripheral axons and ultimately death of SGNs (Kujawa and Liberman, 2009). The consequences of synaptic and neuronal loss are deficits in hearing acuity, leading to difficulty in speech recognition and listening in noisy environments (Liberman, 2017). This type of auditory dysfunction is referred to as noise-induced cochlear synaptopathy (NICS) or hidden hearing loss (HHL) because it can precede hair cell dysfunction or loss and is not readily diagnosed by standard clinical hearing tests such as auditory brainstem response (ABR) thresholds or distortion product otoacoustic emission (DPOAE) levels. Notably, Veterans reporting high levels of military noise exposure display reduced suprathreshold ABR peak I amplitudes (input/output neural function), which has been used as a functional read-out for cochlear synaptopathy (Bramhall et al., 2017). Currently, there are no FDA-approved drugs that elicit regeneration of lost SGNs and restore their synaptic connections with the surviving hair cells. Moreover, loss of SGNs can limit the effectiveness of primary therapies for hearing loss, such as hearing aids and cochlear implants or any future hair cell regeneration therapies. Neurotrophins such as NT-3, BDNF, or agonists for Trk receptor (Neurotrophin receptor) can partially regenerate synapses in pre-clinical animal models of NICS (Wan et al., 2014; Suzuki et al., 2016; Hashimoto et al., 2019; Chen et al., 2018; Fernandez et al., 2021). However, such partial effectiveness clearly underscores a need to further delineate the cellular and molecular mechanisms of SGN survival and synapse repair or regeneration in order to identify newer targets and develop therapies to fully restore loss of synapses and hearing in NICS.

We have established a novel and critical role for cochlear macrophages (innate-immune cells) and fractalkine signaling in synaptic repair and SGN survival after NICS (Kaur et al., 2019; Manickam et al., 2023). The fractalkine signaling axis (CX_3_CL1–CX_3_CR1) involves a unique neuron-immune ligand–receptor pair in which fractalkine ligand (CX_3_CL1 or FKN), a chemokine, is constitutively expressed on neurons in the central nervous system (CNS) (Harrison et al., 1998; Kim et al., 2011) and on mouse SGNs and IHCs (Kaur et al., 2015) and human SGNs (Liu et al., 2018). FKN is the exclusive ligand of a G-protein-coupled receptor, CX_3_CR1, which is expressed by both human and mouse microglia (brain resident macrophages) (Jung et al., 2000) and cochlear resident macrophages (Hirose et al., 2005). FKN is the only member of the CX3C, or delta, chemokine subfamily (Bazan et al., 1997; Mizoue et al., 2001). Unlike most other chemokines, FKN is a 395 amino acid (aa) type I transmembrane protein (Stothert and Kaur, 2021), including a signal sequence (aa 1–24), a chemokine domain (aa 25–100), a mucin stalk region (aa 101–336), a transmembrane segment (aa 337–357), and a cytoplasmic tail (aa 358–395). FKN exists in two different forms: a membrane-bound protein (mFKN) tethered to neuronal membranes by a mucin-like stalk and a soluble factor (sFKN) released upon cleavage of its N-terminal chemokine domain by metalloproteinases (Imai et al., 1997; Garton et al., 2001). The soluble chemokine domain of fractalkine acts as a chemoattractant, promoting migration of immune cells expressing CX_3_CR1, while the membrane-tethered mucin stalk of fractalkine acts as an adhesion molecule for leukocytes to endothelium during tissue injury (Haskell et al., 1999; Hermand et al., 2008). Pre-clinical animal studies in the CNS have shown that neurotransmitter glutamate-induced excitotoxic injury activates and recruits microglia to the site of injury, and that those microglia protect neurons and improve synaptic recovery following excitotoxic damage (Berezovskaya et al., 1995; Vinet et al., 2012; Simard and Rivest, 2007; Eyo et al., 2014; Kato et al., 2016). Such microglia-mediated protection against CNS excitotoxicity has been partly attributed to fractalkine signaling (Meucci et al., 1998; Chapman et al., 2000; Deiva et al., 2004; Limatola et al., 2005; Ragozzino et al., 2006; Lauro et al., 2010; Lauro et al., 2008; Cipriani et al., 2011; Catalano et al., 2013; Roseti et al., 2013; Cardona et al., 2006). Moreover, administration of the soluble isoform of FKN (sFKN) confers neuroprotection, synaptic recovery, and improved function in animal models of several neurodegenerative and neuroinflammatory disorders (Noda et al., 2011; Pabon et al., 2011; Morganti et al., 2012; Nash et al., 2013; Nash et al., 2015; Qin et al., 2014; Limatola and Ransohoff, 2014; Mendiola et al., 2016; Finneran et al., 2019; Wang et al., 2019; Gayen et al., 2022; de Almeida et al., 2023; Jong et al., 2023; Lauro et al., 2015).

We have demonstrated in mice that noise trauma that induces a temporary shift in hearing thresholds (TTS) without any significant hair cell damage and/or loss causes rapid degeneration of IHC ribbon synapses and migration of resident cochlear macrophages into the damaged IHC synaptic region (Kaur et al., 2019). We have shown in mice (Kaur et al., 2019; Manickam et al., 2023) and others have reported in zebrafish, mice, rat, guinea pigs, and gerbils (Puel et al., 1995; Zheng et al., 1997; Wang and Green, 2011; Shi et al., 2015; Shi et al., 2013; Kim et al., 2019; Hickman et al., 2020; Hickman et al., 2021; Jeffers et al., 2021; Holmgren et al., 2021) that noise-damaged IHC synapses can undergo spontaneous repair/regeneration. Our research has also shown that genetic disruption of fractalkine signaling due to lack of CX_3_CR1 receptor on macrophages or absence of cochlear resident macrophages hampers spontaneous synaptic repair and augments SGN loss and inflammation after noise trauma (Kaur et al., 2019; Manickam et al., 2023; Kaur et al., 2018). These results suggest that endogenous intact fractalkine signaling axis plays a key role in synaptic repair and SGN survival through suppression of inflammation in the noise-damaged cochlea. Given the critical role of fractalkine signaling in synaptic repair, it is important to understand its impact on spontaneous repair mechanisms. Here, we investigated whether boosting the cochlear FKN levels exogenously is adequate to restore loss of synapses and hearing after NICS. Our data serve as a proof-of-principle that local delivery of an immune factor, sFKN peptide, is effective in restoring the loss of IHC ribbon synapses and hearing after NICS, and sFKN-mediated synaptic repair is dependent on cochlear macrophages. They also demonstrate that transtympanically delivered sFKN peptide localizes to the sensory epithelium and resolves inflammation due to NICS. These findings highlight the potential of sFKN as an immune-related therapeutic target for cochlear synaptopathy due to noise.

## Materials and methods

### Mice

The study used in-house bred age-matched FKN wild-type mice (hereafter denoted as FKN WT) (Jackson Laboratories, Bar Harbor, Maine, stock number, 000664) and CX_3_CL1^−/−^ (FKN knockout) mice (hereafter denoted as FKN KO) (Cook et al., 2001), gifted by Dr. Astrid Cardona, University of Texas, Department of Molecular Microbiology and Immunology, College of Sciences, San Antonio, Texas. FKN KO mice were bred to congenicity to the parental C57BL/6 J strain (~13 backcrosses) in Dr. Cardona’s laboratory before they were transferred. Efforts were made to minimize animal suffering and to reduce the number of animals used. Animals were housed under a 12-h light/12-h dark cycle and fed *ad libitum*, unless specified. All aspects of animal care, procedure, and treatment were carried out according to guidelines of the Animal Studies Committee of Creighton University, Omaha, NE and Rutgers University, Piscataway, NJ.

### Chemicals and peptides

Chemicals and peptides were purchased from Fisher Scientific, Sigma-Aldrich, or R&D systems until mentioned otherwise. Poloxamer 407 was purchased from Sigma-Aldrich (cat. # 16758). Recombinant control peptide (R&D systems, cat. # 4460, Accession # P01863) is sourced from a mouse melanoma cell line, NS0-derived mouse IgG2A protein of 26.7 kDa and 232 amino acids. Recombinant membrane-bound Fractalkine (R&D systems, cat. # 472-FF, Accession # O35188) is sourced from *Spodoptera frugiperda*, sf21 (baculovirus)-derived mouse CX3CL1/Fractalkine protein of 34 kDa and 312 amino acids. Recombinant soluble Fractalkine (R&D systems, cat. # 571-MF, Accession # AAB71763) is sourced from *E. coli*-derived mouse CX3CL1/Fractalkine peptide of 80 amino acids (aa 25–105) of 9.3 kDa. All peptides used in the study were free of bovine serum albumin (BSA) as a stabilizer.

### Transtympanic (TT) injection

Lyophilized powders of the peptides indicated above were reconstituted with sterile 1× PBS, pH 7.4 (Fisher Scientific, cat. # BP661-10) to an equimolar stock concentration, aliquoted, and stored at −80°C until needed for experiment. Immediately before the TT injection, peptide aliquots were mixed with an equal volume of sterile 18% poloxamer 407 hydrogel, which was prepared in PBS to prolong the residence time of the injected peptide in the middle ear (Salt et al., 2011). Mice were anesthetized with an intraperitoneal injection of Ketamine/Xylazine (Patterson Veterinary) cocktail (100 and 20 mg/kg, respectively, dose, 0.1 mL/20 g mouse body weight), following procedures described in our previous publications (Kaur et al., 2019). An anesthetized mouse’s ear was brought under the focus of a stereo dissection microscope (Vermont Optechs, Inc., Maine, USA). A speculum was placed in the outer ear. A pressure-release hole was carefully made in pars tensa of the tympanic membrane using 1 mL insulin syringe needle (29 gage, Excel, cat. # 26029). The injection hole of ~0.5 mm was made ~90° angle opposite to the exhaust hole below the pars flaccida. Vehicle or peptide mixed with vehicle was injected by inserting the needle (31 gage, Hamilton, cat. # 7762–04) attached to a 10 μL Hamilton syringe into the injection hole and the contents were slowly released near the round window. The mouse was placed on a heating pad with the injected ear up until it recovered from the anesthesia. In order to initially examine the ability of FKN peptides in ribbon synapse repair following NICS, all peptides were TT injected at a higher concentration of 50 ng/μl [modified based on the reported EC_50_ in CNS studies (Noda et al., 2011; Pabon et al., 2011; Morganti et al., 2012; Nash et al., 2013; Nash et al., 2015; Qin et al., 2014; Limatola and Ransohoff, 2014; Mendiola et al., 2016; Finneran et al., 2019; Wang et al., 2019; Gayen et al., 2022)] in a total injection volume of 5 μL (total peptide delivered = 250 ng). All TT injections were performed once and unilaterally.

### Study design

**Figure 1:**
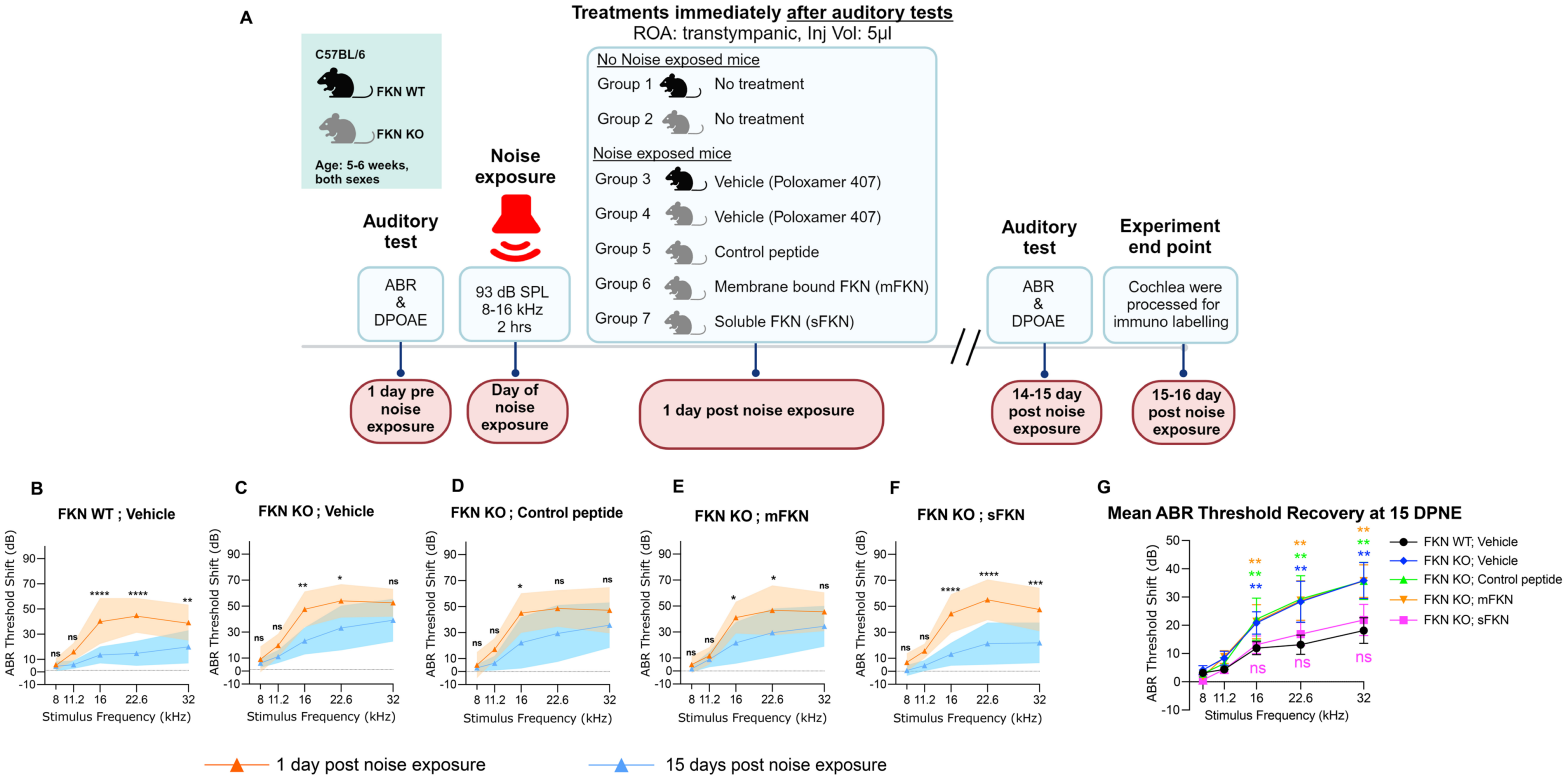
ABR threshold shifts at 1 DPNE and 15 DPNE. (A) Study design created with BioRender.com. ROA, Route of Administration. (B–F) ABR threshold shifts at 1-day post-noise exposure (DPNE) and 15 DPNE after 2 h at 93 dB SPL at 8–16 kHz octave band noise in (B) FKN WT mice treated with vehicle (*N* = 8), and in FKN KO mice treated with (C) vehicle (*N* = 6) (D) control peptide (*N* = 7) (E) membrane-bound FKN peptide (mFKN) (*N* = 9) and (F) soluble FKN peptide (sFKN) (*N* = 8). Dashed line represents threshold shifts prior to noise exposure (baseline). Values are means ± SD. *p < 0.05, **p < 0.01, ***p < 0.001, ****p < 0.0001, and ns, non-significant at respective stimulus frequency. *Represents comparison between 1 DPNE and 15 DPNE, two-way ANOVA, Sidak’s multiple comparisons. (G) Mean ABR threshold shift recovery at 15 DPNE of all the experimental groups. Values are means ± SD. **p < 0.01 and ns, non-significant at respective stimulus frequency. *Represents comparison between FKN WT; vehicle (black circle) versus all other treatment groups, two-way ANOVA, Dunnett’s multiple comparisons.

Both male and female FKN WT and KO mice 5–6 weeks of age were used for the study. To avoid the saturation effect of the cochlear endogenous FKN ligand, FKN KO mice were used to test the efficacy of FKN isoforms in restoration of ribbon synapses after NICS. Following assessment of baseline hearing sensitivity at 1-day pre-noise exposure (PNE), mice were exposed to a moderate synaptopathic noise level of 93-dB sound pressure level (dB SPL) at 8–16 kilohertz (kHz) for 2 h (Manickam et al., 2023). Exposure to 93 or 94 dB SPL results in a significant IHC ribbon synapse loss in the basal region of the cochlea that does not undergo spontaneous repair, thereby allowing the examination of the ability of administration of FKN peptides in restoration of damaged ribbon synapses after NICS (Kaur et al., 2019; Manickam et al., 2023; Kim et al., 2019; Wu et al., 2024). The degree of hearing loss was measured at 1-day post-noise exposure (DPNE), immediately after which the noise-exposed FKN WT mice were TT injected with the vehicle (sterile 18% Poloxamer 407 hydrogel prepared in PBS). Noise-exposed FKN KO mice were injected either with vehicle (18% Poloxamer 407 hydrogel), control peptide, membrane-bound FKN peptide (mFKN), or soluble FKN peptide (sFKN). The degree of recovery of hearing threshold shifts was measured at 14–15 DPNE, following which the mice were euthanized and isolated cochleae were microdissected and processed for various assays indicated below. Age-matched, unexposed, and uninjected FKN WT and FKN KO mice served as controls (Figure 1A).

### Noise exposure

Awake, unrestrained mice were exposed for 2 h to an octave band noise (8–16 kHz) at noise levels of 93 dB SPL. Noise exposures were performed in a foam-lined, single-walled soundproof room (WhisperRoom Inc., TN). Briefly, mice were placed singly or in pairs in modified subdivided cages (food, water, and bedding removed) and positioned up to two cages at once directly under an exponential horn. Noise was generated digitally using custom Labview routines and a Lynx E22 sound card (running on a PC), filtered pure tone (8–16 kHz), and amplified (D-150A power amplifier, Crown Audio) that drive the speaker through an exponential horn. Prior to each exposure, the noise was calibrated to target sound pressure levels (SPL) using a quarter-inch condenser microphone (PCB). Sound pressure levels varied by ±1 dB across the subdivisions in the cage. Unexposed mice served as age-matched controls.

### Electrophysiological recordings

Auditory brainstem responses (ABR) and distortion product otoacoustic emissions (DPOAE) were measured by an observer who was “blinded” to the experimental conditions of each animal. Mice were anesthetized with an intraperitoneal injection of Ketamine/Xylazine (Patterson Veterinary) cocktail (100 and 20 mg/kg, respectively, dose, 0.1 mL/20 g body weight), following procedures described in our previous publications (Kaur et al., 2019). ABR thresholds and DPOAE levels were measured prior to noise exposure, 1 day after noise exposure (to verify degree of hearing loss), and 2 weeks after noise exposure, to quantify recovery of hearing.

#### ABRs

Anesthetized mice were placed on a heating pad set at 37°C, and eyes were lubricated with an ophthalmic ointment (artificial tears) to avoid drying due to anesthesia. Subcutaneous needle electrodes were placed behind the right pinna (reference) and vertex (active). A ground electrode was placed near the tail of the mouse. A TDT Multi-Field (MF1) speaker was set at 10 cm from the right pinna and calibrated with PCB ¼″ free field calibration microphone before recordings. Stimuli of 5-ms tone pips (0.5 ms cos^2^ rise-fall) were delivered at 21/s with alternating stimulus polarity using an RZ6-A-P1 Bioacoustic system and Medusa4Z pre-amplifier (Tucker-Davis Technologies, Alachua, FL). Recorded electrical responses at a sampling rate of 12 kHz were filtered (300 Hz to 3 kHz) and averaged using BioSigRZ software (Tucker-Davis Technologies, Alachua, FL). The sound level was decreased in 5-dB steps from 100 dB Sound Pressure Level (SPL) down to threshold. At each sound level, 512–1,024 responses were averaged, and response waveforms were discarded as artifacts if the peak-to-peak voltage exceeded 15 mV. Thresholds at 8.0, 11.2, 16.0, 22.6, and 32.0 kHz were determined by a single observer who noted the lowest sound level at which a recognizable Peak I (PI) waveform could be obtained. Waveforms were confirmed as auditory-evoked responses by their increasing latency and decreasing amplitude as the intensity of the stimulus was lowered. ABR thresholds refer to the lowest SPL that can generate identifiable wave I. These threshold values (actual or assigned) were used to calculate the mean ABR thresholds at each stimulus frequency. Threshold shifts were calculated by subtracting baseline thresholds from thresholds at 1-day and 15-day post-noise exposure at all the stimulus frequencies.

#### Neural input/output (I/O) response

Methods followed those described in Kaur et al. (2019). Briefly, ABR Peak I component was identified, and the peak-to-trough amplitudes and latencies were computed by off-line analysis of stored ABR waveforms. ABR Peak I amplitude and latency versus stimulus level (ABR I/O) data were computed at 8, 16, and 32 kHz for sound levels ranging from 100 dB SPL up to the hearing thresholds, unless otherwise specified.

#### DPOAEs

Anesthetized mice were placed on a heating pad set at 37°C, and eyes were lubricated with an ophthalmic ointment (artificial tears) to avoid drying due to anesthesia. Stimuli were presented at 5–40 kHz and delivered to the right ear by a custom coupling insert RZ6-A-P1 bioacoustic system (Tucker-Davis Technologies, Alachua, FL). Electrostatic loudspeakers (EC1) for generating f1 and f2 were calibrated with ER10B+ etymotic low-noise probe and microphone before recordings. Distortion product (DP) grams (2f1–f2) were obtained for f2 ranging from 5 to 40 kHz, with a frequency ratio of f2/f1 of 1.2 and L1–L2 = 10 dB. The L1 and L2 were set at 75 and 65 dB SPL, respectively. Recordings were performed using BioSigRZ software (Tucker-Davis Technologies, Alachua, FL). Following ABR/DPOAE recordings, animals were placed on a heating pad at 37°C and monitored until they regained activity and then returned to animal research facility.

### PLX5622-induced depletion of cochlear macrophages

To deplete cochlear macrophages, PLX5622 was used, which is a selective antagonist of colony stimulating factor 1 receptor (CSF1R) that regulates macrophage development, survival, and maintenance (Elmore et al., 2014). Briefly, FKN WT mice were fed on either standard nutritionally complete rodent diet (AIN-76A formulation, referred to as control chow) or on AIN-76A chow that contained 1.2 g/kg of PLX5622 (hemifumarate salt, 99.30% purity, cat. # HY-114153, MedChemExpress, NJ, referred to as PLX5622 chow). Both control and PLX5622 chows were formulated, irradiated, and supplied by Research Diets, Inc., New Brunswick, NJ. Following 2 weeks on respective chows, which led to a robust elimination of cochlear resident macrophages, both control and PLX5622-fed FKN WT mice were exposed to noise after recording the baseline hearing sensitivity, followed by the TT injection of either vehicle or sFKN peptide at 1 DPNE, and the special chows were maintained until the end of the experiment for sustained macrophage depletion. Hearing function was assessed again at 15 DPNE, followed by euthanasia and processing of isolated cochleae for synaptic immunolabeling. Feeding mice with PLX5622 chow for 15 days does not affect the normal hearing sensitivity, as shown in our previous work (Manickam et al., 2023).

### Tissue harvesting and processing

Mice were deeply anesthetized with lethal doses of pentobarbital sodium (Trade Name-Fatal Plus, Patterson Veterinary). Prior to respiratory arrest, mice were perfused by transcardiac route with phosphate-buffered saline (PBS) (Fisher Scientific, cat. # BP661-10) or 4% paraformaldehyde (Fisher Scientific, cat. # 50980495) in 0.1 M phosphate-buffered solution, and temporal bones were harvested. For fluorescent immunolabeling of cochlear synapses, excised temporal bones were post-fixed in 4% paraformaldehyde for 20 min on ice and decalcified in 0.1 M ethylenediaminetetraacetic acid (EDTA, Fisher Scientific, cat. # AC327205-000) overnight for ~16 h. For macrophage immunolabeling in cochlear mid-modiolar cryosections, temporal bones were fixed for 3–4 h at room temperature and decalcified for 3–5 days. The temporal bones were rinsed three times with PBS, and appropriate fluorescent immunohistochemistry was performed.

### Immunohistofluorescence

Microdissected cochlear whole mounts or frozen mid-modiolar cross sections (20 mm) were rinsed with PBS (Fisher Scientific, cat. # BP661-10) at least 3 times and incubated at room temperature for 2 h in blocking solution containing 5% normal horse serum (Sigma-Aldrich, cat. # H0146) in 0.2% Triton X-100 (Sigma-Aldrich, cat. # A16046AP) in PBS. For synaptic immunolabeling, cochlear microdissected whole mounts were incubated overnight at room temperature with combinations of the following primary antibodies: CtBP2 mouse (BD Biosciences, cat. # 612044; RRID:AB_399431; 1:200), GluA2 mouse (EMD Millipore, cat. # AB1506; RRID:AB_11212990; 1:100), Myosin 7a rabbit (Proteus Biosciences, cat. # 25–6,790; RRID:AB_2314838; 1:500). For macrophage and neuron immunolabeling, cochlear frozen mid-modiolar cross sections or whole mounts were incubated overnight at room temperature with combinations of the following primary antibodies: goat anti-CD45 (R&D Systems, cat. # AF114; RRID:AB_442146; 1:100), mouse anti-Neurofilament 165 (NF-H) (Developmental Studies Hybridoma Bank, cat. # 2H3C, 1:100), and mouse anti-Tuj1 (Covance, cat. #MMS-435P, 1:500). Following incubation with primary antibodies, specimens were rinsed 5 times in PBS and treated for 2 h at room temperature in species-specific secondary antibodies conjugated to either DyLight 405 (Jackson ImmunoResearch Laboratories Inc., 1:500) or AlexaFluor-488, −546, −555, or −647 (Invitrogen, Life Technologies, 1:500). Tissue was mounted in glycerol:PBS (9 parts glycerol and 1 part PBS) and coverslipped before confocal imaging. Tissue samples were batch-processed using the same reagent solutions for all experimental groups.

### Confocal imaging

Three- or four-color fluorescence imaging was performed using a Zeiss LSM 700 laser scanning confocal microscope (Carl Zeiss Microscopy 700, Jena, Germany). *Z*-series images using 5×, 10×, 20×, 40×, or 63× objectives were obtained. Image processing and quantitative analyses were performed using IMARIS (version 9.9.0, Oxford Instruments) and Volocity 3D image (version 6.5.1, PerkinElmer) software.

### Synapse count

Three confocal *z*-stacks were obtained using a high-resolution oil immersion objective (63× with 1.4 numerical aperture) and a digital zoom of 1.5 from apical (~4, 8, and 12 kHz), middle (~16, 20, and 24 kHz), and basal (~28, 32, and 40 kHz) regions per cochlear whole mount. Each *z*-stack spanned the entire synaptic pole of 8–10 IHCs in the *z*-dimensions, with *z*-step-size of 0.3 μm, from the apical portion of the IHC to nerve terminal in the habenula perforata region. IMARIS was used for 3D analysis of individual and juxtaposed/paired pre- and postsynaptic puncta counts. Thresholds for all the channels were adjusted in such a way the background is reduced without losing any prominent fluorescence and two close fluorescent puncta are not merged. Total CtBP2 puncta, total GluA2 puncta, and paired CtBP2 and GluA2 fluorescence surface were counted per image. Total counts were divided by the number of surviving IHC to report CtBP2 puncta per IHC, GluA2 puncta per IHC, and paired ribbon synapses per IHC.

### Macrophage count

To evaluate the effect of FKN on macrophage density, CD45 (macrophage marker in the cochlea)-labeled macrophages were counted in the sensory epithelium from the apical, middle, and basal regions of cochlear whole mounts of unexposed and noise-exposed FKN WT and FKN KO mice as previously described in Kaur et al. (2019), Manickam et al. (2023), and Kaur et al. (2015). Briefly, following intensity thresholding, CD45 fluorescence intensity was automatically quantified by IMARIS software and was verified manually from 40× objective images (2–3 images) per region per cochlea. Raw macrophage counts were normalized to the length of the sensory epithelium and averaged as macrophages per 100 μm of sensory epithelium.

### ELISA

Protein extracted from cochlear lysate was estimated using a BCA kit (Thermo Scientific, cat. # 23225). Level of cochlear endogenous FKN (R&D systems, # DY472) or TT-injected (exogenous) sFKN peptide (Invitrogen, cat. # EMCX3CL1) was estimated from 50 μg of cochlear protein lysate by following the manufacturer’s protocol. Briefly, cochlear protein lysates (samples) were loaded along with the sFKN standard in a 96-well plate coated with capture antibody. Plate was incubated for 150 min at room temperature. After washing, the wells were equally treated with binding antibody, followed by biotin conjugate, streptavidin-HRP, and TMB substrate. The yellow color developed at the end point was read at 450 nm using Varioskan LUX multimode plate reader (Thermo Fisher Scientific, # VLBL0TD2). Concentration of sFKN in cochlear protein lysate was determined using the linear equation derived from the standard curve (Supplementary Figure S12A).

### Matrix assisted laser desorption ionization-time of flight-mass spectrometry (MALDI-TOF-MS)

A cohort of FKN KO mice were TT-injected with sFKN peptide, and cochlear perilymph (~0.5 μL) was collected according to procedures described in (Salt et al., 2006; Swan et al., 2009) at 1, 3, 6, 12, and 24 h after injection (a different mouse was used for perilymph collection at each indicated time point). Each sample was diluted with 4.5 μL of mass spectrometry (MS) grade water (with 0.1% trifluoroacetic acid) to prepare final volume of 5 μl. A total 1 μL of each diluted perilymph sample was spotted on a MALDI stainless steel plate, and 1 μL insulin as internal standard (9 ng/μl) along with 1 μL of DHB matrix solution (40 mg/mL) in acetonitrile: water 1:1 (0.1% TFA) were added on the same spot for each time point. For a standard curve, sFKN peptide was dissolved in MS grade water (with 0.1% TFA) at 250 ng/μl and diluted sequentially two times up to 0.48 ng/μl, spotted on the MALDI plate in triplicate, and then 1 μL insulin as internal standard (9 ng/μl) and 1 μL of DHB matrix solution were then added to each spot.

The samples were dried at room temperature. MALDI-TOF-mass spectroscopy experiments were performed in an Autoflex III MALDI-TOF/TOF mass spectrometer (Bruker Daltonics, Leipzig, Germany) equipped with a 200-Hz Smart beam laser and using the Flex control v.3.4 software. Mass data were collected with manual laser positioning. For each sample, 10 spots were chosen, and 1,000 laser shots were collected to obtain 10 spectra in linear positive mode. The IS1 voltage was 20 kV, the IS2 voltage was maintained at 18.97 kV, the lens voltage was 5.67 kV, and laser power was 95%. Sample rate and digitizer setting were set to 1.25 and detector gain was 10×. Mass data were collected in the range 5,000–14,000 m/z. Mass accuracy was calibrated internally with spiked insulin, and the intensity of internal standard and sFKN were determined from each sample. Standard curve was prepared by plotting intensity ratio of sFKN/insulin in *Y*-axis and concentration of peptide in *X*-axis and fitting the data with linear equation (Supplementary Figure S12B). Mass of peptide in perilymph was then calculated from standard curve equation.

### Fluorescent conjugated sFKN peptide detection in the cochlea after TT injection

Mouse sFKN Alexa Fluor-647 (Almac, # CAF-51, 9.9 kDa) was reconstituted in PBS, and an equimolar amount of the unconjugated sFKN peptide (i.e., 50 ng/μl) was mixed with sterile Poloxamer 407 hydrogel and TT injected in FKN WT mice. The cochlea and perilymph were isolated at 3- and 6-h post-injection. Mid-modiolar cryosections of the cochlea were processed for macrophage immunolabeling as described above and imaged using confocal microscope. The collected perilymph was made up to 50 μL in 1× PBS, and the fluorescence intensity or emission was measured at 647 nm using Varioskan LUX multimode plate reader (Thermo Fisher Scientific, # VLBL0TD2).

### Luminex assay

Luminex assay was performed to detect the protein levels of cytokines in cochlear protein lysates using a multiplex panel as per the manufacturer’s protocol (Thermo Fisher Scientific, cat. # PPX-26-MXPRMA9). The panel includes cytokines and chemokines such as IL-1*α*, IL-1β, IL-2, IL-4, IL-6, IL-10, IL-12, IL-13, IL-15, IL-17, IL-18, IL-22, IL-23, IL-33, M-CSF, IFNα, IFNβ, IFNγ, TNF-α, CXCL1, CXCL2, CXCL10, CCL2, CCL3, CCL4, and CCL7. Briefly, magnetic beads coated with target antibodies were added to the 96-well assay plate and washed three times with wash buffer. Standards for 26 cytokine and chemokine analytes were mixed and serially diluted to generate standard curves. Cochlear protein lysates at a concentration of 50 μg were used. Antigen standards, blanks, and sample lysates were incubated with magnetic beads at room temperature at 500 rpm for 120 min in the dark. Then beads were washed three times to remove the excess lysate/standard. A total 25 μL of detection antibody was added and incubated in the dark for 30 min at room temperature. The beads were washed three times, 50 μL of streptavidin-PE was added, and incubated for 30 min in the dark at room temperature. Washed beads were re-suspended in 120 μL of reading buffer, and the plate was read using a Luminex™ 100/200™ system (Thermo Fisher Scientific, FLEXMAP 3D). Acquired raw data were analyzed using the 5PL algorithm offered by Thermo Fisher Scientific (Procartaplex Analysis App available online). Raw data files from the instrument were fed in the application, and the lot numbers of standards were entered. By following the manufacturer’s instructions, the application computes the concentration and displays the graph for individual analytes. From the panel of 26 cytokines, those that were significantly changed after noise trauma and returned to baseline after sFKN treatment were selected for data presentation.

### Statistical analyses

All statistical analyses were performed using Prism GraphPad versions 10.0.2 and 10.2.0. Values are expressed as mean ± standard deviation (SD) across animals in each experimental group unless otherwise stated in the figure legends. Student’s *t*-test, or one- or two-way ANOVA, was applied as appropriate. Significant main effects or interactions were followed by appropriate *post-hoc* tests. Details on error bars, statistical analysis, numbers of mice, and experimental replicates can be found in the results and figure legends. Results were considered statistically significant when the probability (*p*-values) of the appropriate statistical test were less than or equal to the significance level, alpha (*α*) = 0.05.

## Results

### sFKN is more effective than mFKN in recovering ABR threshold shifts after NICS in FKN KO mice

Our overarching goal in this study was to evaluate the efficacy of FKN isoforms (membrane-bound and soluble) in restoring IHC ribbon synapses and hearing after NICS. We first assessed the efficacy of FKN isoforms in the recovery of ABR thresholds after NICS. Gross hearing function and IHC ribbon synapse density in unexposed FKN KO mice were comparable to the age-matched unexposed WT mice (Supplementary Figure S1). At 1 DPNE, both FKN WT and KO mice showed a similar degree of elevation in ABR thresholds, with FKN KO mice displaying ~8–10 dB higher threshold shifts (not significant, *p* = 0.965, 0.968, 0.633, 0.399, and 0.100 at 8, 11.2, 16, 22.6, and 32 kHz, respectively, two-way ANOVA, Sidak’s multiple comparison test) than FKN WT mice compared to the thresholds before noise exposure (Figures 1B,C; Supplementary Figure S2). There is an average 40 and 50 dB shift in ABR thresholds at stimulus frequencies of 16 kHz and above at 1 DPNE in the FKN WT and KO mice, respectively. By 15 DPNE, vehicle-treated WT mice showed significant recovery in ABR thresholds to baseline (Figures 1B,G; Supplementary Figure S2). By contrast, similar threshold improvement was not observed in the vehicle-treated FKN KO mice (Figures 1C,G; Supplementary Figure S2), suggesting that endogenous FKN may have a protective role in influencing the recovery of hearing thresholds after a moderate synaptopathic noise trauma. Remarkably, this loss of hearing function in the noise-exposed FKN KO mice was significantly rescued by the single transtympanic administration of sFKN peptide, and the degree of recovery was comparable to the recovery observed in the vehicle-treated FKN WT mice (Figures 1F,G *P* = 0.9734, two-way ANOVA, FKN WT; Vehicle vs. FKN KO; sFKN). By comparison, the recovery in ABR thresholds in the noise-exposed FKN KO mice injected with either control peptide (Figures 1D,G; Supplementary Figure S2) or mFKN peptide (Figures 1E,G; Supplementary Figure S2) was comparable to the vehicle-treated FKN KO mice and was significantly different from vehicle-treated FKN WT or sFKN peptide-treated FKN KO mice (*p* = 0.0042, two-way ANOVA, FKN WT; Vehicle vs. FKN KO; Control peptide, *p* = 0.0030, two-way ANOVA, FKN WT; Vehicle vs. FKN KO; mFKN and *p* > 0.999, two-way ANOVA, FKN KO; Vehicle vs. FKN KO; Control peptide or mFKN). While both the genotype and treatment with peptides influenced the ABR threshold improvement at 15 DPNE, there was no significant difference observed in the DPOAE levels at 15 DPNE when compared to pre-noise exposure for each of the experimental groups, suggesting that there is no significant effect of either genotype or treatment with peptides on the outer hair cell function (Supplementary Figure S3). Furthermore, the absence of FKN did not influence the survival of inner and outer hair cells in both unexposed and noise-exposed mice (Supplementary Figure S4).

### sFKN is more effective than mFKN in restoring ABR peak I amplitudes after NICS in FKN KO mice

**Figure 2:**
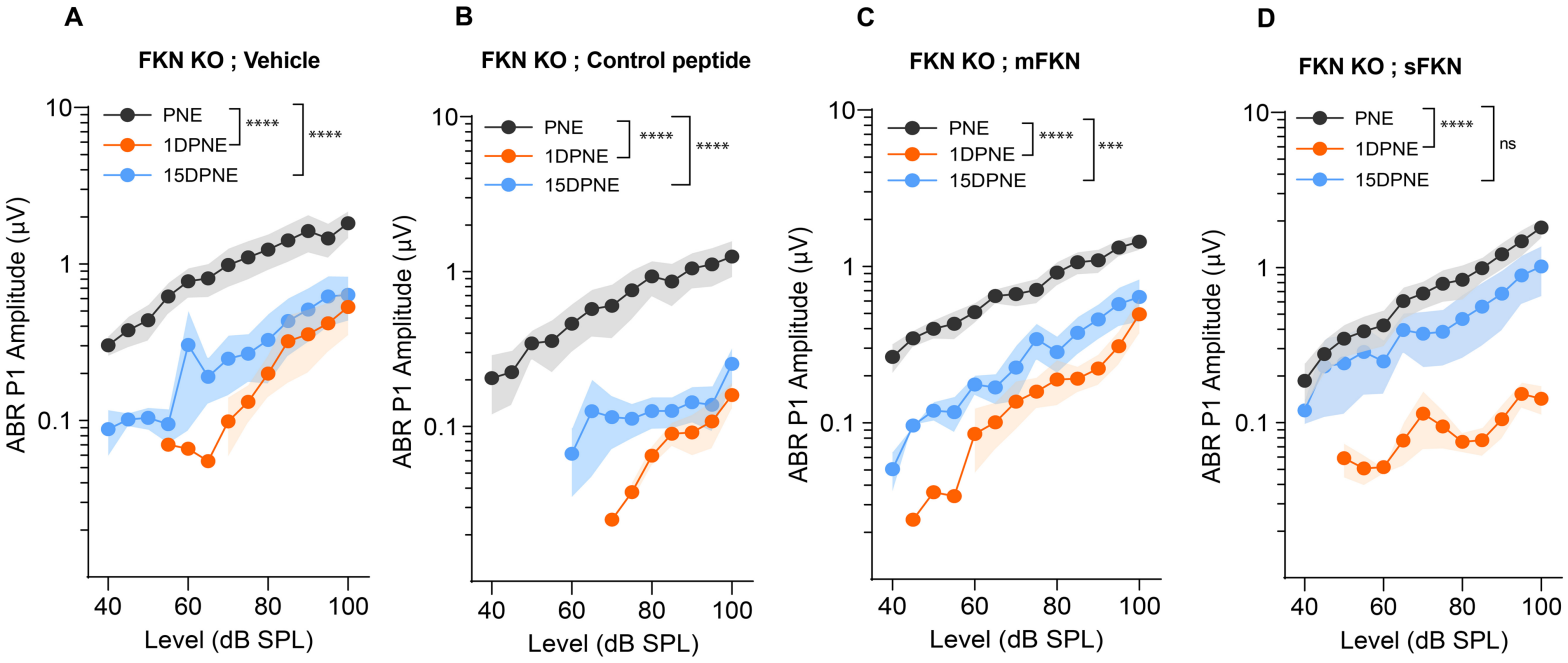
ABR peak I amplitude in FKN KO mice. ABR peak I amplitudes at 32 kHz at pre-noise exposure (PNE), 1 DPNE, and 15 DPNE in FKN KO mice treated with (A) vehicle (*N* = 6) (B) control peptide (N = 7) (C) membrane-bound FKN peptide (mFKN) (*N* = 10) and (D) soluble FKN peptide (sFKN) (*N* = 8). Values are means ± SD. ***p < 0.001, ****p < 0.0001, and ns, non-significant. The symbol * represents the comparison between the experimental time points as indicated with parenthesis. One-way ANOVA, Tukey’s multiple comparison test.

Next, we measured ABR peak I amplitudes to determine which of the FKN isoforms (membrane-bound and soluble) is effective in recovering the function of IHC ribbon synapses after NICS. At 32 kHz, suprathreshold responses were significantly reduced in FKN KO mice by 1-day post-synaptopathic noise exposure (Figure 2). None of the FKN KO mice injected with either vehicle, control peptide, or mFKN peptide showed recovery in ABR-PI amplitude by 15 DPNE (Figures 2A–C). Nevertheless, FKN KO mice injected with sFKN peptide displayed near complete recovery (~85%) of ABR-PI amplitudes to baseline by 15 DPNE (Figure 2D). Similarly, at 22.6 kHz, suprathreshold response recovered in FKN KO mice injected with sFKN peptide by 15 DPNE when compared to FKN KO mice that were injected with either vehicle, control peptide, or mFKN (Supplementary Figure S5). Collectively, these and the above data implicate endogenous FKN in regulating the recovery of threshold sensitivity. Furthermore, administration of sFKN peptide in mice lacking endogenous FKN is more effective than mFKN peptide in restoring ABR-PI amplitude after NICS. No significant difference was observed in ABR-PI amplitudes at 8 and 16 kHz, where initial shifts were small, between sFKN treatment and other experimental groups (Supplementary Figure S5). Similarly, ABR-PI latencies at 8, 16, and 32 kHz were largely unaffected among the experimental groups at all recovery time points after noise trauma (Supplementary Figure S6). These results suggest that there is no effect of either genotype or treatment on ABR-PI amplitudes and latencies at these stimulus frequencies.

### sFKN is more effective than mFKN in restoring noise-damaged ribbon synapses in FKN KO mice

**Figure 3:**
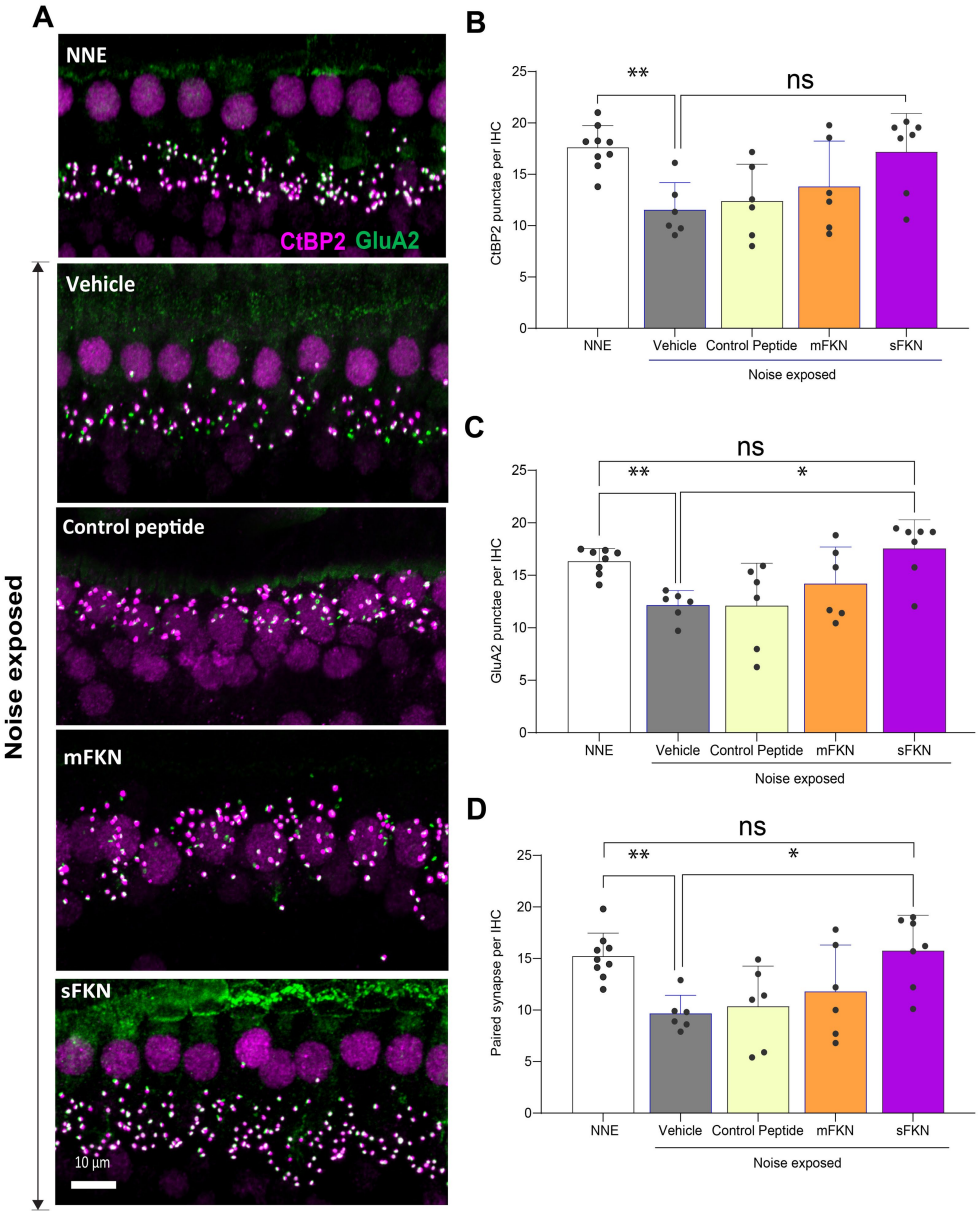
Inner hair cell ribbon synapses in basal cochlear region of FKN KO mice. (A) Representative micrographs showing IHC-paired ribbon synapses in the basal cochlear region after 15 days of synaptopathic noise exposure. (B) CtBP2 puncta per IHC. (C) GluA2 puncta per IHC. (D) Paired ribbon synapses per IHC. Values are mean ± SD. Each dot in the graphs represents one mouse. Three confocal images were captured from the basal cochlear region per mouse. CtBP2 puncta, GluA2 puncta, and paired synapses per IHC were averaged across the three images per mouse and plotted. **p < 0.01 between no noise-exposed (NNE) vs. noise-exposed vehicle-treated FKN WT and FKN KO mice; *p < 0.05 between noise-exposed FKN KO mice treated with vehicle or sFKN peptide; ns: non-significant between NNE and NE sFKN-treated FKN KO mice. One-way ANOVA, Dunnett’s multiple comparison test. *N* = 5–9 mice per experimental group.

Next, we investigated if local administration of sFKN peptide restores noise-damaged ribbon synapses. To address this, microdissected cochleae were immunolabeled for presynaptic CtBP2 ribbons and postsynaptic GluA2 AMPA receptors (Figure 3A). At 15 DPNE, no significant loss was observed with CtBP2 puncta, GluA2 puncta, and paired ribbon synapses per IHC in apical and middle cochlear regions in all the noise-exposed experimental groups (Supplementary Figure S7). Compared to no noise-exposed FKN KO mice, there was a significant reduction in the CtBP2 puncta (does not display ribbon “survival”, i.e., counts normalized to mean control value) (Figure 3B), GluA2 puncta (Figure 3C), and paired synapses (Figure 3D) per IHC (from ~18 to ~10 puncta or synapses per IHC) in the basal region of the cochlea of noise-exposed FKN KO mice at 15 DPNE. Among the noise-exposed FKN KO groups, mice injected with sFKN peptide showed a significant restoration of GluA2 puncta and paired synapses per IHC (from ~10 to ~17 puncta or synapses per IHC) when compared to mice injected with mFKN peptide or with control peptide (Figures 3C,D). CtBP2 puncta per IHC was not improved significantly with sFKN peptide treatment when compared to noise-exposed vehicle-treated FKN KO group (Figures 3B; *P* > 0.999, one-way ANOVA, Dunnett’s multiple comparison test). Furthermore, such synaptic repair due to sFKN peptide treatment yields IHC synaptic density comparable to no noise-exposed FKN KO group (*p* = 0.990, one-way ANOVA, Dunnett’s multiple comparison test) and positively correlates with the recovery of ABR peak I input/output neural response at 32 kHz (Figure 2D). This and above data suggest that between the two FKN isoforms, sFKN is more efficacious in restoring damaged synapses in noise-exposed FKN KO mice, both structurally and functionally.

### Single TT injection of sFKN peptide restores noise-damaged ribbon synapses and ABR-PI amplitude in FKN WT mice

**Figure 4:**
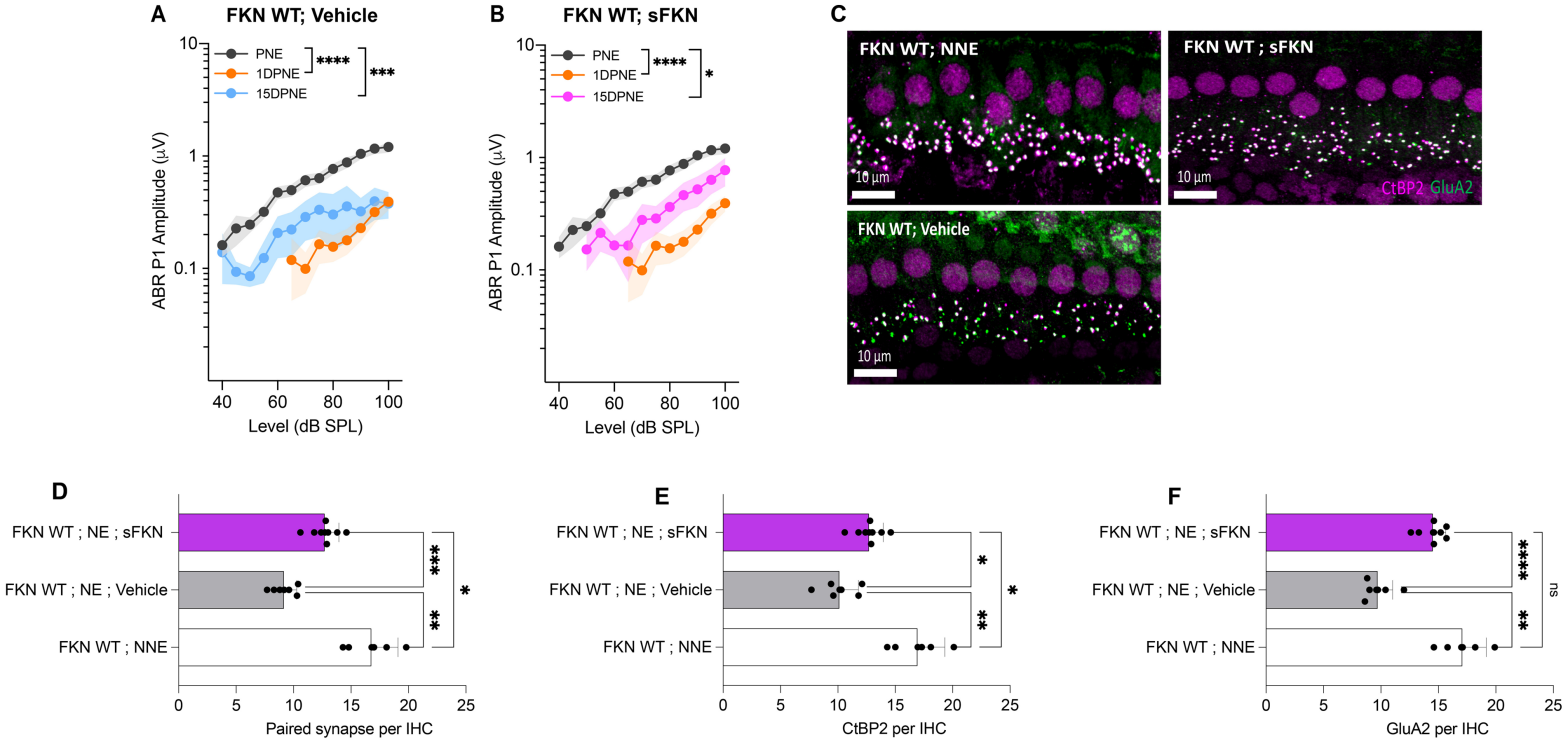
ABR peak I amplitude and IHC ribbon synapse density in FKN WT mice. (A) ABR peak I amplitude at 32 kHz at pre-noise exposure (PNE), 1 DPNE, and 15 DPNE in FKN WT mice treated with vehicle. (B) ABR peak I amplitude at 32 kHz at pre-noise exposure (PNE), 1 DPNE, and 15 DPNE in FKN WT mice treated with a single dose of sFKN peptide. *N* = 15 mice (PNE and 1 DPNE), *N* = 8 mice (15 DPNE) in A and B. (C) Representative micrographs showing IHC ribbon synapses from basal cochlear region at 15 DPNE. (D) Paired ribbon synapses per IHC. (E) CtBP2 puncta per IHC. (F) GluA2 puncta per IHC. Values are means ± SD. N = 5–8 mice per experimental group. Each dot in graphs (D–F) represents one mouse. *p < 0.05, **p < 0.01, ***p < 0.001, ****p < 0.0001, and ns, non-significant. One-way ANOVA, Tukey’s multiple comparison test.

Although threshold sensitivity recovered in vehicle-treated FKN WT mice (Figure 1A), suprathreshold response at 32 kHz (high-frequency region), where acute threshold shifts were large, had not recovered (Figure 4A), displaying the hallmark of noise-induced hidden hearing loss (Kujawa and Liberman, 2009). The input/output data show significant reduction in ABR-PI amplitudes at suprathreshold sound levels at 1 DPNE and 15 DPNE compared to baseline (Figure 4A). Similarly, compared to unexposed mice, there was a significant reduction in paired synapses (Figures 4C,D), CtBP2 puncta (Figures 4C,F), and GluA2 puncta (Figures 4C,F) per IHC (from ~18 to ~9 puncta or synapses per IHC) in the basal region of the cochlea of noise-exposed vehicle-treated FKN WT mice at 15 DPNE. Since there appeared no spontaneous synaptic repair in the basal cochlear region of the noise-exposed vehicle-treated FKN WT mice, we wondered whether this is likely due to the inadequate levels of cochlear endogenous FKN ligand naturally promoting synaptic repair after NICS. Accordingly, cochlear endogenous levels of sFKN were measured by ELISA in FKN WT mice at different days after NICS, and there was only a small increase in the levels relative to the control unexposed cochlea (Supplementary Figure S8). Therefore, this result prompted us to exogenously administer sFKN peptide in the noise-exposed FKN WT mice to boost FKN levels to test whether sFKN peptide restores noise-damaged ribbon synapses in FKN WT mice. We administered sFKN peptide in FKN WT mice at 1-day post-noise exposure and analyzed ABR-PI amplitudes at 32 kHz (Figure 4B) and IHC ribbon synapse density in the basal cochlear region (Figures 4C–F). In contrast to vehicle-treated FKN WT mice, ABR-PI amplitudes significantly recovered in FKN WT mice injected with sFKN peptide by 15 DPNE (Figure 4B). Moreover, noise-damaged IHC ribbon synapses were found to be restored as a result of treatment with sFKN peptide (from ~9 to ~13 synapses per IHC) (Figure 4D), with significant improvement in GluA2 puncta (Figure 4F) than CtBP2 puncta (Figure 4E) per IHC. Such synaptic recovery positively correlated with the ABR-PI recovery in sFKN-treated wild-type mice. Together, these data suggest that the administration of sFKN peptide can attenuate synaptic damage both functionally and structurally in the wild-type mice besides FKN KO mice. Since mFKN peptide and control peptide did not demonstrate any significant synaptic and functional recovery in FKN KO mice after NICS, they were not tested in FKN WT mice.

### Cochlear macrophages are indispensable for sFKN-mediated IHC synaptic repair after NICS

**Figure 5:**
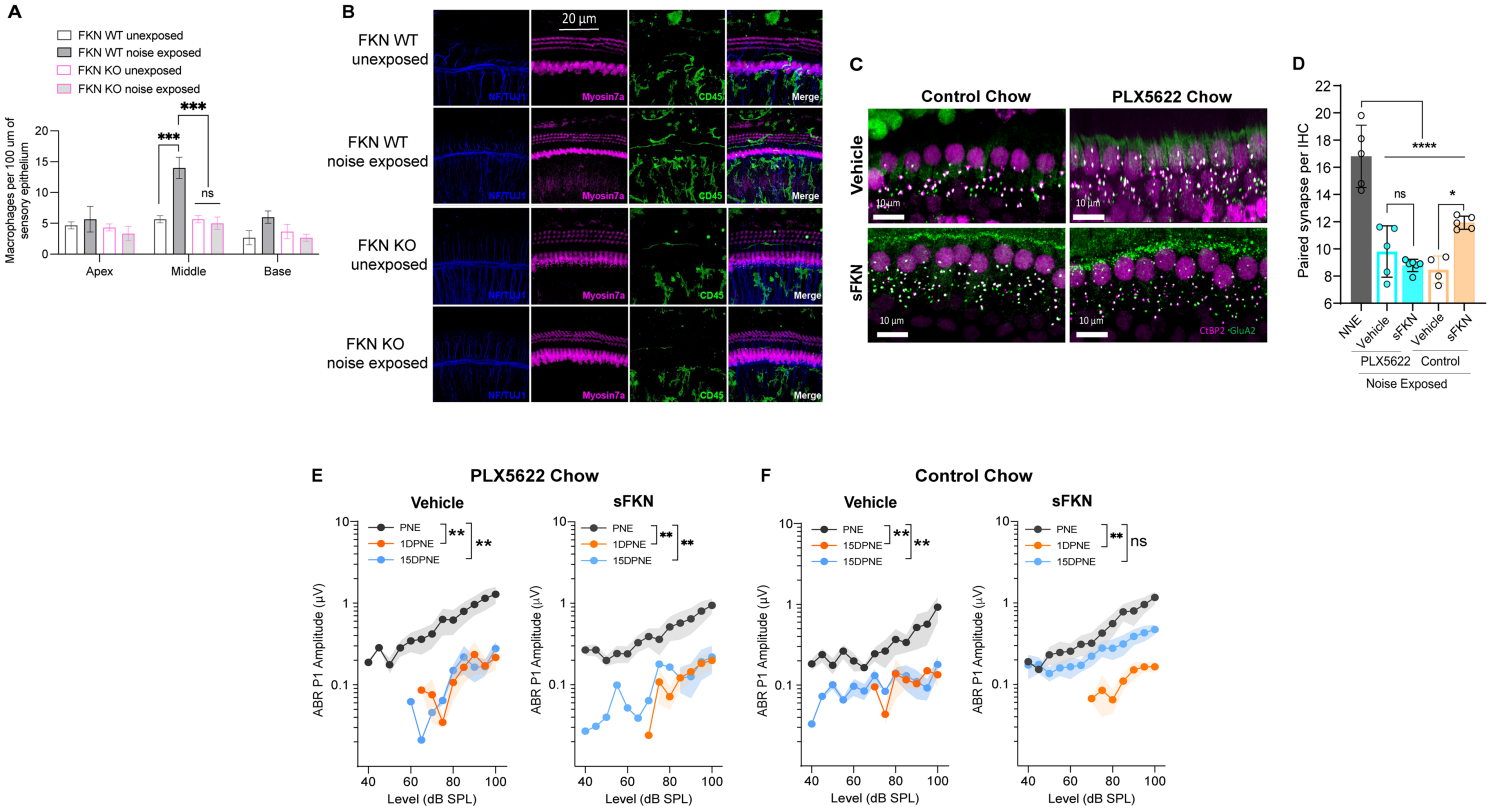
sFKN fails to restore noise-damaged IHC ribbon synapses and ABR Peak I amplitudes in FKN WT mice lacking cochlear resident macrophages. (A) Macrophage density in the sensory epithelium of apex, middle, and basal cochlear regions of unexposed and noise-exposed FKN WT and FKN KO mice. Two-way ANOVA, Tukey’s comparison test. *N* = 3 mice per genotype. (B) Representative micrographs showing CD45-immunolabeled macrophages in the sensory epithelium of the middle cochlear region of unexposed and noise-exposed FKN WT and FKN KO mice. (C) Representative micrographs showing IHC-paired ribbon synapses from the basal cochlear region of vehicle- or sFKN peptide-treated FKN WT mice in the presence (control chow) or absence (PLX5622 chow) of macrophages at 15 DPNE. (D) Quantification of paired ribbon synapses per IHC in vehicle- or sFKN peptide-treated FKN WT mice in the presence (control chow) or absence (PLX5622 chow) of macrophages at 15 DPNE. Gray bar represents unexposed (NNE) FKN WT mice data re-represented from Figure 4C for comparison purposes and to reduce the use of mice as per IACUC policies. Two-way ANOVA, Tukey’s comparison test. (E,F) ABR Peak I amplitudes at 32 kHz in vehicle- or sFKN peptide-treated FKN WT mice in the absence [PLX5622 chow (E)] or presence [control chow (F)] of macrophages at 15 DPNE. One-way ANOVA, Dunnett’s multiple comparison test. *N* = 5–7 mice per experimental group (D,E). Values are means ± SD in D or means ± SEM in (E,F). *p < 0.05, **p < 0.01, ***p < 0.001, ****p < 0.0001, and ns, non-significant.

We previously reported that cochlear resident macrophages expressing CX_3_CR1 are activated and migrate immediately into the sensory epithelium in close proximity to the damaged IHC synaptic region following a synaptopathic noise trauma (Kaur et al., 2019). To determine whether chemokine FKN influences macrophage localization and numbers in the damaged sensory epithelium after NICS, cochlear whole mounts were immunolabeled for CD45, a pan-leukocyte marker that labels CX_3_CR1-expressing cochlear macrophages (Hirose et al., 2005). The macrophage numbers increased in the middle (~2.5-fold) and basal (~2-fold) cochlear region of the damaged sensory epithelium of FKN WT mice after noise trauma when compared to unexposed FKN WT mice. However, such an increase in macrophage numbers in the sensory epithelium was not observed in the noise-damaged FKN KO mice (Figures 5A,B). There was no significant change in macrophage numbers in the apical region of the cochlea in either noise-exposed FKN WT or KO groups compared to unexposed mice. This implies that chemokine, FKN, which is endogenously expressed by the IHCs, supporting cells, and SGNs (Kaur et al., 2015), may regulate macrophage numbers in the sensory epithelium in response to NICS.

FKN binds to its sole receptor, CX_3_CR1, which is thought to be specifically expressed by macrophages (Hirose et al., 2005). This fact, and data indicating that both FKN (Figures 5A,B) and CX_3_CR1 (Kaur et al., 2015; Kaur et al., 2018) regulate macrophage density in the damaged cochlea, prompted us to ask whether the sFKN-mediated synaptic repair is a direct effect of sFKN on IHC ribbon synapses and SGNs, or does it require cochlear macrophages expressing CX_3_CR1. To determine whether macrophages are necessary for sFKN-mediated synaptic repair after NICS, cochlear macrophages were depleted in FKN WT mice via treatment with CSF1-R inhibitor, PLX5622 chow for 2 weeks, which effectively resulted in ~94% ablation of cochlear resident macrophages (Manickam et al., 2023) (also see Supplementary Figure S9). Following macrophage ablation, noise-exposed FKN WT mice were injected with either vehicle or sFKN peptide, and synapses and function were analyzed at 15 DPNE. Depletion of cochlear macrophages with PLX5622 abrogated the ability of sFKN peptide to restore noise-damaged ribbon synapses (Figures 5C,D) and ABR Peak I amplitudes (Figure 5E) in the basal cochlear region of the FKN WT mice. By contrast, sFKN peptide, but not vehicle treatment, restored the loss of synapses and function in the basal cochlear region in the presence of cochlear resident macrophages (control chow) in FKN WT mice (Figures 5C,D,F, also see Figure 4). The present data also corroborate our previous report (Manickam et al., 2023) that the absence of cochlear macrophages impairs the improvement of ABR thresholds, which is not affected by sFKN peptide treatment, whereas in the presence of macrophages elevated ABR thresholds recover with or without sFKN treatment (Supplementary Figures S9A–D). DPOAE levels were comparable among the experimental groups at all recovery time points (Supplementary Figures S10E–H). Mechanistically, these data argue that sFKN peptide restores noise-induced loss of IHC ribbon synapses and functions in a macrophage-dependent manner.

### NICS-induced pro-inflammatory cytokines are diminished by sFKN

**Figure 6:**
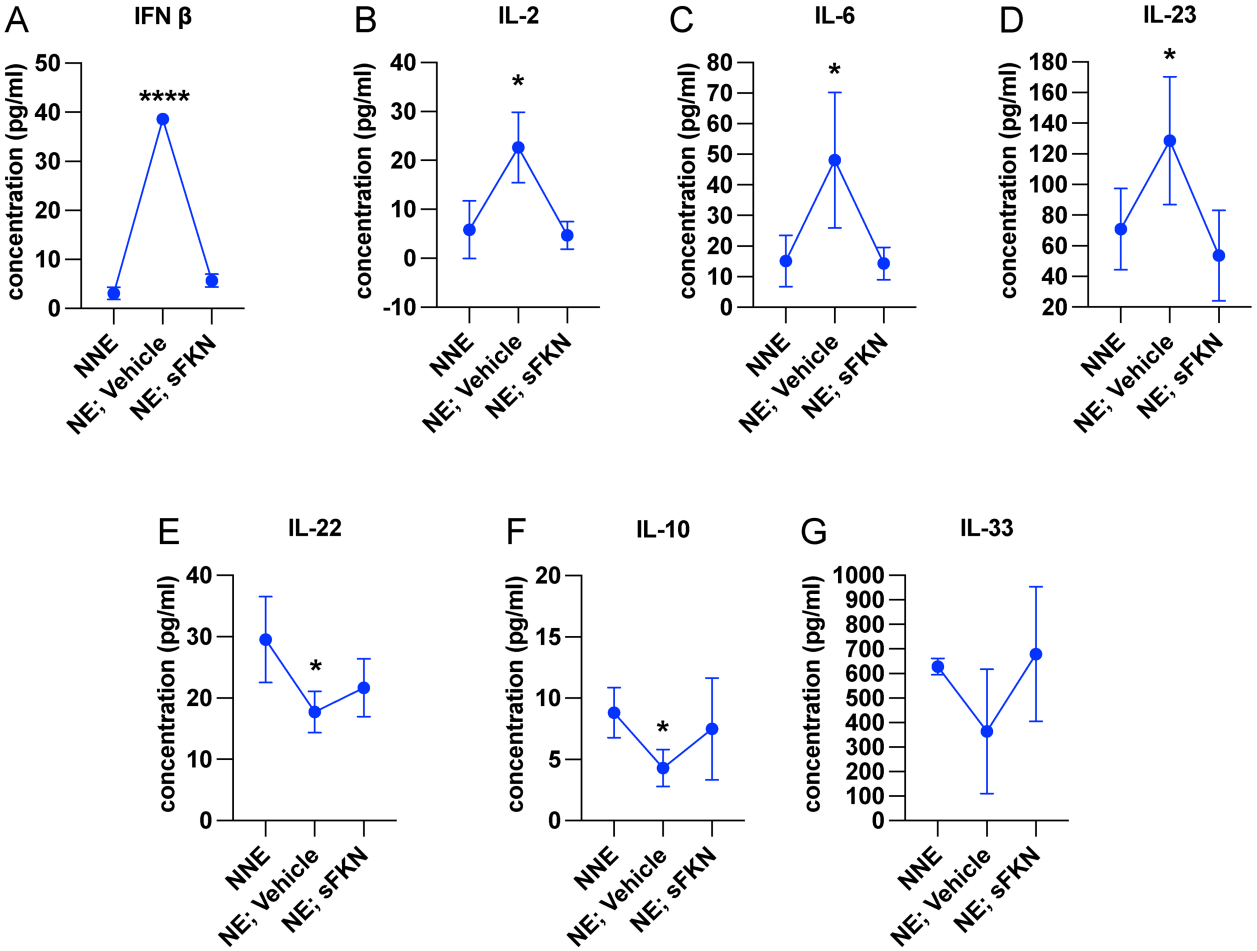
sFKN peptide modulates cochlear inflammation profile after NICS. Luminex assay-based quantification of the levels of cytokines (A) IFN-*β*, (B) IL-2, (C) IL-6, (D) IL-23, (E) IL-22, (F) IL-10, and (G) IL-33 in cochlear lysate from FKN WT mice subjected to either no-noise (NNE) or noise-exposed, then TT injected with vehicle at 1-day post-exposure (NE; vehicle) or noise-exposed and TT injected with sFKN peptide at 1-day post-exposure (NE; sFKN). *N* = 3–4 biological replicates per experimental group. Each biological sample was a pool of five cochleae run in triplicate. Values are means ± SD. *p < 0.05, ****p < 0.001, NNE vs. NE; vehicle or NE; sFKN vs. NE; vehicle. There was no significant difference in the means between NNE and NE; sFKN experimental groups. One-way ANOVA, Dunnett’s multiple comparison test.

Cochlear synaptopathy is associated with activation of macrophages (Kaur et al., 2019) and upregulation of inflammation (Wu et al., 2020). Since CX_3_CL1 signaling has been shown to attenuate the production of pro-inflammatory cytokines and decrease activation of macrophages *in vitro* and *in vivo* following various inflammatory stimuli (Harrison et al., 1998; Ransohoff et al., 2007; Maciejewski-Lenoir et al., 1999; Garcia et al., 2000; Zujovic et al., 2000; Zujovic et al., 2001; Re and Przedborski, 2006; Lyons et al., 2009; Mizutani et al., 2007), we examined whether sFKN administration regulates cochlear inflammation in response to NICS in the FKN WT mice. Cochlear protein lysates from non-noise-exposed mice (NNE), noise-exposed mice receiving vehicle at 1-day post-exposure (NE; vehicle), and noise-exposed mice receiving sFKN peptide 1-day post-exposure (NE; sFKN) were collected at ~48-h post-noise exposure and analyzed for cytokines levels using the Luminex-based multiplex technique. Because cochlear inflammation resolved by 15 days after such an acute noise exposure (data not shown), this time point was not taken into consideration to examine the ability of sFKN peptide to regulate cochlear inflammation after NICS. The Luminex data show that the production of pro-inflammatory cytokines such as IFN-*β*, IL-2, IL-6, and IL-23 was significantly elevated in the cochleae injected with vehicle at 1 day after a synaptopathic noise trauma compared to non-noise-exposed FKN WT mice. However, treatment with sFKN peptide attenuated or maintained the levels of these cytokines similar to the levels found in the non-noise-exposed FKN WT mice (Figures 6A–D). Similarly, levels of anti-inflammatory cytokines such as IL-10, IL-22, and IL-33 were reduced in the cochleae injected with vehicle 1 day after a synaptopathic noise trauma compared to non-noise-exposed FKN WT mice. However, treatment with sFKN peptide after noise trauma elevated or maintained cytokine levels close to baseline (Figures 6E–G). We also applied RT-qPCR to measure messenger RNA (mRNA) levels for cochlear CX_3_CR1 receptor in FKN WT mice following injection of sFKN peptide. No substantial change was detected in the expression of CX_3_CR1 mRNA among the unexposed, noise-exposed vehicle-injected, or noise-exposed sFKN injected groups at 2- and 24-h post-sFKN peptide injection (Supplementary Figure S11). Collectively, these results suggest that sFKN-mediated IHC synaptic repair after NICS is likely attributable to the regulation of cochlear inflammation by sFKN in response to NICS.

### sFKN peptide is bioavailable in the cochlea following transtympanic injection

**Figure 7:**
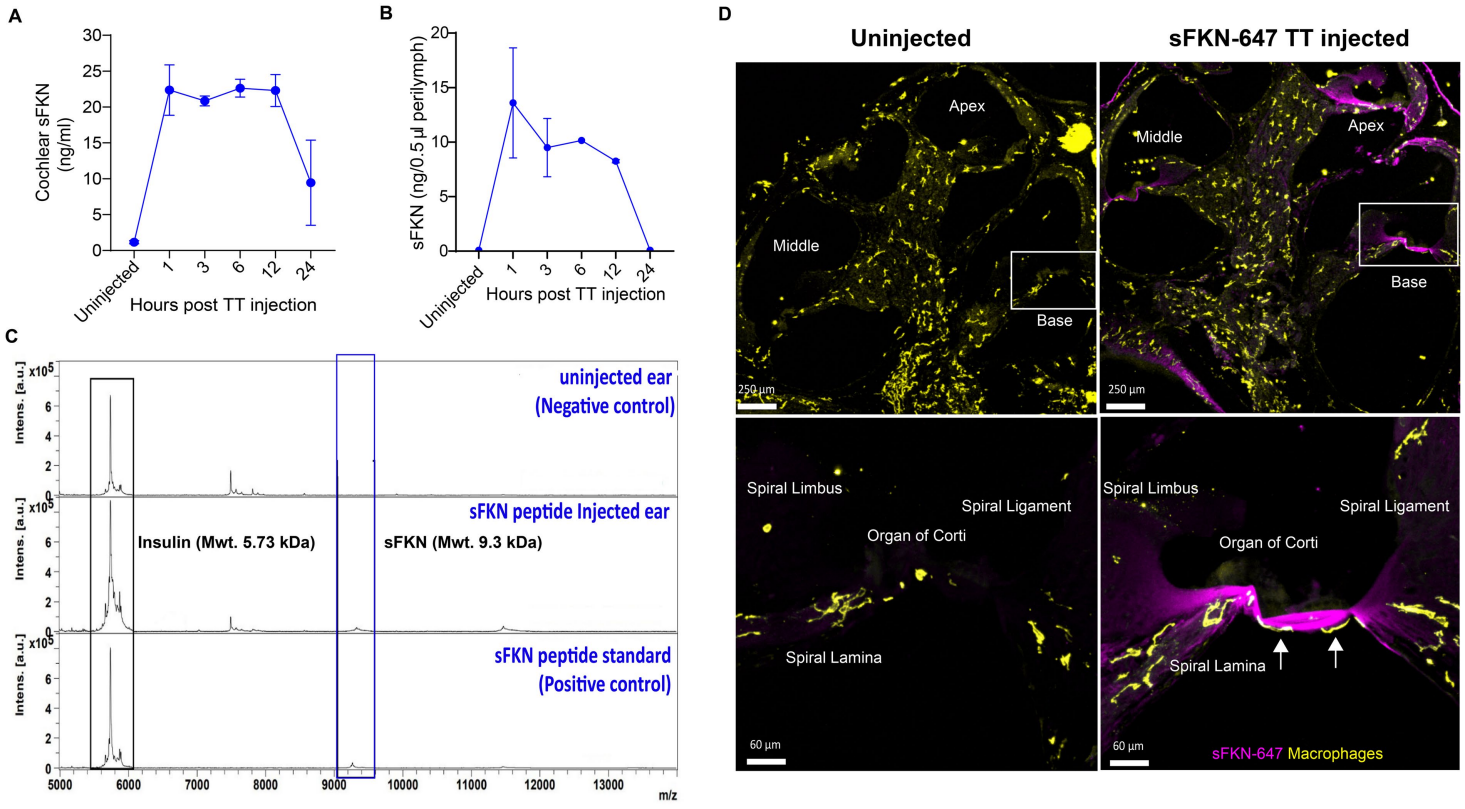
Temporal and spatial bioavailability of sFKN peptide after TT injection in FKN KO and FKN WT mice. Estimation of levels of sFKN peptide in (A) cochlear protein lysate by ELISA and (B) cochlear perilymph by MALDI-TOF-MS at different time points after TT injection in FKN KO mice. Values are means ± SD. *N* = 2 biological replicates per time point after TT injection and uninjected group. (C) Representative MALDI-TOF mass spectrometric peaks of sFKN peptide (9.3 kDa) at 3 h after injection in FKN KO mice when compared to uninjected ear (blue bar). Peaks in the bottom panel represent that of the standard sFKN peptide of 9.3 kDa as positive control. Peaks of the left represent that of insulin peptide as internal control (black bar). (D) *Upper panel*, representative images of cochlear mid-modiolar cross section at low magnification. *Bottom panel*, representative images at higher magnification of the basal cochlear turn (white rectangular box in upper panel) showing the localization of fluorescent conjugated sFKN-647 (magenta) to the basilar membrane near the sensory epithelium, spiral limbus, osseous spiral lamina, and spiral ligament of uninjected and injected FKN WT mice. CD45-immunolabeled macrophages (white arrows) are found to be adhered to the undersurface of the basilar membrane among the mesothelial cells in the injected cochlea.

To verify that the TT-injected sFKN peptide into the middle ear reaches the inner ear, cochlear protein lysate and perilymph were extracted at different time points after sFKN injection in FKN KO mice, and sFKN level (cochlear bioavailability) was estimated by ELISA (Figure 7A) and MALDI-TOF-MS (Figures 7B,C), respectively. Compared to the uninjected or control ears, a significant level of sFKN was detected 1 h after injection in cochlear protein lysate by ELISA (Figure 7A) and in perilymph by MALDI-TOF-MS (Figure 7C). Approximately 1/10–1/25 of the total sFKN peptide (250 ng in 5 μL at a concentration of 50 ng/μl) injected transtympanically into the middle ear appeared to reach sites inside the cochlea. The sFKN level in the cochlea was maintained for 12-h post-injection after which the level sharply declined by 24-h post-injection. Additionally, mouse sFKN conjugated to Alexa Fluor 647 (sFKN-647) was injected in FKN WT mice to visualize the spatial distribution of sFKN-647 at 3-h post-injection. Data show that injected sFKN-647 is localized to the basilar membrane near the sensory epithelium, spiral lamina, spiral ligament, and to the collagen fibrils in the spiral limbus in all three turns at 3-h post-injection (Figure 7D, right panel) compared to the uninjected ear (Figure 7D, left panel). Similarly, elevated mean fluorescence intensity of conjugated sFKN-647 in cochlear perilymph was detected 3 h after injection (Supplementary Figure S13). CD45 immunolabeled cochlear macrophages were also seen to be adhered to the undersurface of the basilar membrane among the mesothelial cells in the injected ear (Figure 7D, bottom right panel) compared to the uninjected ear (Figure 7D, bottom left panel). Collectively, these data from complementary approaches confirm that sFKN peptide is bioavailable in the cochlea for up to 24 h after TT injection and is localized near the sensory epithelium and to the spiral lamina, where it appears positioned to restore the noise-damaged IHC ribbon synapses and function.

## Discussion

Mature (Signal Sequence-free) FKN is a 373-aa protein with a single transmembrane domain that can undergo proteolytic cleavage to release sFKN into the extracellular environment (Bazan et al., 1997). In the CNS, both mFKN and sFKN are primarily produced by neurons, and by binding CX_3_CR1 on microglia, they are thought to regulate key aspects of microglial physiology (Lauro et al., 2015; Paolicelli et al., 2014). One of the main tasks of FKN in neuron–microglia interactions is to suppress the activation of microglia (Zujovic et al., 2000; Mizuno et al., 2003). Supporting this notion, exogenous delivery of sFKN has been shown to decrease microglia activation as well as neurological deficits in animal models of Parkinson’s disease, stroke, retinitis pigmentosa, and diabetic retinopathy (Cipriani et al., 2011; Pabon et al., 2011; Nash et al., 2015; Mendiola et al., 2016; Wang et al., 2019). In the cochlea, FKN is constitutively expressed by sensory SGNs, IHCs, and certain supporting cells (Kaur et al., 2015), and endogenous sFKN is released after selective cochlear hair cell ablation (Kaur et al., 2015), or after NICS (Supplementary Figure S7), and is upregulated after a permanent hearing threshold shift-imparting noise trauma (Milon et al., 2021). Moreover, in the absence of CX_3_CR1 on cochlear macrophages, there is an impairment in spontaneous synaptic repair and enhanced loss of SGNs after NICS (Kaur et al., 2019). Based on these data, we boosted the levels of FKN in the cochlea at 1 day after a synaptopathic noise trauma of 2 h at 93 dB SPL with the hope that it would restore the damaged IHC ribbon synapses and hearing function. Single transtympanic injection of sFKN peptide indeed restored noise-damaged synapses and hearing function, and it did so in the presence of cochlear macrophages while attenuating inflammation due to NICS. Remarkably, synaptic repair was seen with the treatment of sFKN, but not with membrane-bound FKN (mFKN), which strongly corroborates the pre-clinical animal studies in the CNS (Morganti et al., 2012; Wang et al., 2019). Such variation in the recovery effects observed between the administration of soluble versus membrane forms of FKN could be due to differences in the bioavailability of the transtympanically injected peptides. Being a smaller peptide (9.3 kDa) than mFKN peptide (34 kDa), sFKN may more efficiently cross the round and/or oval window and be better able to reach cochlear resident macrophages. Of note, using three complementary approaches (i.e., ELISA, MALID-TOF-MS, and conjugated sFKN-647) we found that the injected sFKN peptide reaches the cochlea and appears bioavailable for up to 24-h post-injection. From there, it putatively ligates to CX_3_CR1 receptor on cochlear resident macrophages to increase their numbers (Figure 7D) and activate downstream signaling, thereby restoring synapses by 2 weeks of post-noise. Alternatively, it could be that ligation with only sFKN but not the mFKN allows for CX_3_CR1 internalization to the cytoplasm following ligation to affect downstream signaling pathways (Morganti et al., 2012; Neel et al., 2005; Borroni et al., 2010). Ligation of mFKN with CX_3_CR1 receptor remains possible through the chemokine domain, but its permanent attachment to the membrane may prevent subsequent internalization to the cytoplasm of macrophages, thereby altering any downstream effectors. Therefore, only the soluble domain of FKN can be readily internalized with CX_3_CR1 ligation. This notion that CX_3_CR1 actively removes sFKN from the surrounding environment is supported by Cardona et al. (2008), where the authors reported substantially more circulating or soluble FKN in CX_3_CR1^−/−^ mice. By using FKN KO mice, we were able to isolate the effects of individual FKN isoform in synaptic repair in response to NICS. sFKN restored noise-damaged ribbon synapses and ABR-PI amplitudes in both FKN KO and WT mice. While synaptic repair was near-to-complete in the FKN KO mice, it was partial in the FKN WT mice. Currently, we do not have a clear understanding of the reasons for this discrepancy between the two genotypes. One possible explanation for this effect could be due to the pharmacological ceiling effect of exogenous sFKN chemokine in the wild-type mice, which also releases endogenous sFKN after NICS to ligate and/or activate the CX_3_CR1 GPCR (Supplementary Figure S8; Pabon et al., 2011). Further studies are required to determine the optimal dosage of sFKN in FKN WT and FKN KO mice for the observed synaptic and functional recovery.

Activated macrophages are associated with damaged ribbon synapses in response to NICS, as evidenced by their immediate migration into the IHC synaptic region, upregulation of inflammatory genes, and phagocytosis of debris (Kaur et al., 2019; Holmgren et al., 2021; Wu et al., 2020). These macrophage activities are beneficial in NICS by promoting IHC synaptic repair, as pharmacological ablation of macrophages has been demonstrated to impair spontaneous synaptic repair (Manickam et al., 2023). Here, we found evidence of macrophage activation during NICS, as illustrated by the presence of macrophages in the sensory epithelium near the IHC synaptic region, consistent with our previous report (Kaur et al., 2019). Interestingly, these macrophages were absent or reduced in numbers in the cochlear sensory epithelium of mice lacking FKN, suggesting a critical role for the endogenous FKN in localizing macrophages to the sensory epithelium in response to NICS. Similarly, more macrophages were localized near the sensory epithelium with the administration of conjugated sFKN-647 peptide in the undamaged cochlea, further supporting the notion that FKN may be a potential chemoattractant for macrophages, at least during acute cochlear injury. Importantly, FKN contributes to wound healing by recruiting macrophages to the site of injury in a mouse model of skin excision (Ishida et al., 2008). Notably, depletion of cochlear resident macrophages abrogated sFKN-mediated restoration of IHC ribbon synapses and function. This indicates that sFKN restores synapses after NICS in a macrophage-dependent manner. We have previously reported that CX_3_CR1, specifically expressed by macrophages, is necessary for spontaneous repair of IHC ribbon synapses after NICS, as deficiency of CX_3_CR1 has been demonstrated to impair synaptic repair (Kaur et al., 2019). Here, we found that administration of sFKN did not alter the mRNA expression of cochlear CX_3_CR1. Consistent with this, it has been reported that sFKN therapy also does not affect the protein levels of CX_3_CR1 in the brain (Morganti et al., 2012). While sFKN does not alter the levels of CX_3_CR1 receptor, whether CX_3_CR1 activation is critical for sFKN-mediated synaptic repair remains to be determined. Studies in the CNS have demonstrated an upregulation of lysosomal pathways in microglia with sFKN therapy, a prominent feature of activated microglia, suggesting these cells may efficiently digest or phagocytose cellular debris, thus favoring preservation of neurons in neurodegenerative diseases (Wang et al., 2019). Moreover, sFKN therapy attenuates the induction of pro-inflammatory cytokines in microglia; in other words, it represses microglial response, as prolonged or uncontrolled induction can result in chronic inflammation, thereby leading to neurodegeneration (Cardona et al., 2006; Morganti et al., 2012; Rodriguez et al., 2024; Rodriguez et al., 2024). Here, we also found evidence for macrophage activation during NICS, as demonstrated by upregulation of IFN-*β*, IL-6, IL-2, and IL-23 cytokines in whole cochlear protein lysates. Remarkably, expression of sFKN attenuated or maintained the production of these cytokines to a similar level found in the undamaged cochlea. Our data are consistent with the CNS studies and indicate a plausible mechanism by which sFKN restore synapses, although how inflammation disrupts intact synapses or impairs repair, remains to be determined. While our data indicate that macrophages are obligatory for sFKN to mediate synaptic repair, the effect of sFKN on phagocytic or lysosomal activation of cochlear macrophages, and the mechanisms by which sFKN–macrophage interaction contribute to recovery of synapses and function, remain to be elucidated.

Our data provide evidence for the lingering presence of sFKN peptide in the cochlea after a single TT injection into the middle ear. Targeted or local delivery like TT injection is preferable to systemic administration because this can circumvent broader, potentially undesirable, effects of the FKN, such as macrophage activation in peripheral organs or in circulation (Apostolakis and Spandidos, 2013). Local application in turn may ultimately facilitate higher local doses and repeat treatments, if needed. In fact, repeated TT injections of gentamicin antibiotics or steroids are routinely done in clinical practice for treating Meniere’s disease or idiopathic sudden sensorineural hearing loss (Greenberg and Nedzelski, 2010). TT injection is also preferred over invasive round window or semicircular canal delivery, which may promote inflammation, possibly requiring further treatment with steroids. Notably, transtympanically injected sFKN peptide was distributed throughout all the cochlear turns and was specifically localized to the basilar membrane and spiral lamina near the sensory epithelium in each turn where the IHC ribbon synapses are damaged after NICS. Thus, sFKN has direct access to damaged sites. Both ELISA and mass spectrometry data revealed the cochlear bioavailability of sFKN peptide with sustained levels for up to 12 h, after which levels appeared reduced. This reduction in sFKN levels could be due to proteolytic degradation of the peptide by proteases and peptidases present in the inner ear fluids (Lin et al., 2019). Nevertheless, our findings open new therapeutic avenues, including development of better analogs of sFKN peptide that are resistant to proteolytic degradation or small molecule agonists of CX_3_CR1 with improved bioavailability. Gene therapy for sFKN can also be employed to achieve prolonged bioavailability of sFKN (Morganti et al., 2012; Nash et al., 2015; Wang et al., 2019; Jong et al., 2023; Rodriguez et al., 2024; Rodriguez et al., 2024), but cochlear delivery and cell-specific transduction of genes are still challenging and entail a risk for immunosuppression. On the contrary, as peptides are short-acting, their therapeutic efficacy, potency, and toxicity can be tightly regulated versus gene therapy. In addition, modulation of FKN signaling with peptides could provide a new prevention and treatment platform for noise-induced hearing loss since peptides present an opportunity for therapeutic intervention that closely mimics natural pathways. Nonetheless, we consider that reported bioavailability of sFKN in the present study is sufficient to ligate CX_3_CR1 and recruit macrophages to the damaged IHC synaptic region in the sensory epithelium to activate downstream signaling pathways like phagocytosis of the synaptic debris and resolution of inflammation that promotes restoration of noise-damaged ribbon synapses and ABR-PI amplitudes.

Currently, there are no FDA-approved drugs or treatments for NICS and other forms of cochlear synaptopathy, a condition that profoundly affects understanding of speech or leads to other central auditory processing disorders. Therefore, there is a great need to identify novel molecules to develop therapeutic targets to restore synapses and hearing in NICS. Our findings have revealed a novel immune pathway, fractalkine signaling axis, and they provide proof-of-principle that sFKN can be developed as an immunotherapy against cochlear synaptopathy. Clinically, sFKN could be administered either alone or together with neurotrophins like NT-3 or BDNF, which also partially restore synapses in animal models of NICS (Wan et al., 2014; Suzuki et al., 2016; Hashimoto et al., 2019; Chen et al., 2018; Fernandez et al., 2021). Due to the anti-inflammatory and regulating macrophage activation properties, sFKN can also be tested against other forms of sensorineural hearing loss, such as age-related hearing loss and cochlear synaptopathy, that are associated with inflammation, vascular damage, and macrophage dysregulation (Lang et al., 2023).

**Figure 8:**
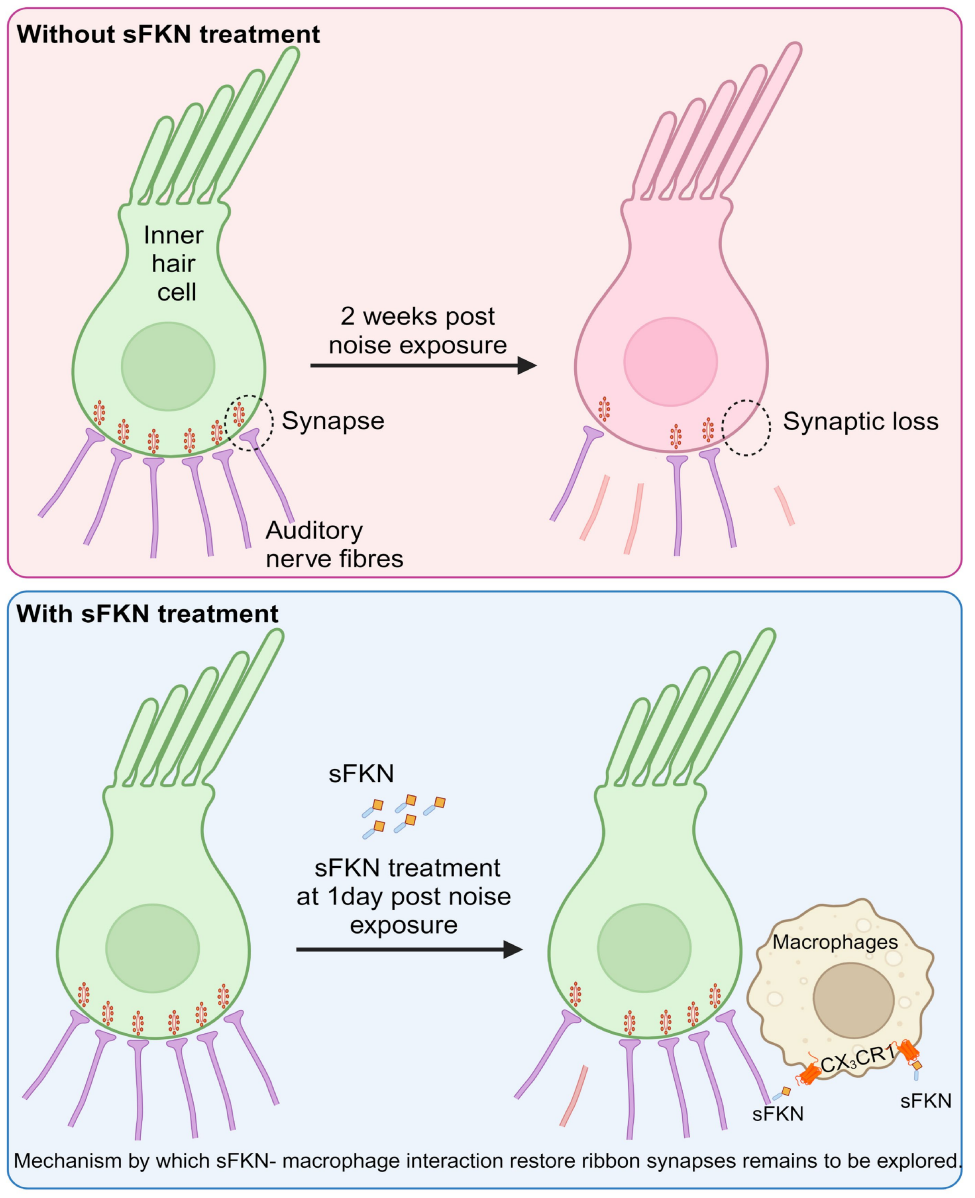
Working model for soluble FKN restores IHC ribbon synapses after NICS. Prolonged or loud exposure to noise results in rapid loss of IHC ribbon synapses known as cochlear synaptopathy. Locally (transtympanically) delivered immune factor, soluble FKN reaches into the cochlea near the sensory epithelium and is effective in restoring the noise-induced loss of IHC ribbon synapses and hearing via cochlear macrophages expressing CX_3_CR1 and suppresses cochlear inflammation in response to noise insult. The precise mechanisms by which sFKN–macrophage interactions contribute to synaptic and functional recovery remain to be elucidated. The figure was created with BioRender.com.

In summary, in this study, we specifically assessed the efficacy of FKN isoforms, i.e., soluble and membrane-bound, in restoration of IHC ribbon synapses and hearing after synaptopathic noise trauma. The present study offers compelling evidence that soluble FKN peptide is more effective than membrane-bound FKN in restoring IHC ribbon synapses and ABR-PI amplitudes in a mouse model of NICS. IHC synaptic repair with sFKN requires the presence of cochlear resident macrophages and is associated with attenuation of inflammation due to NICS without altering the expression of CX_3_CR1 receptor on cochlear macrophages. We also report that sFKN peptide is bioavailable in the cochlea for up to 24 h after a single transtympanic injection and appears localized to the basilar membrane and spiral lamina near the sensory epithelium (Figure 8). While the mechanisms by which sFKN–macrophage interaction restore ribbon synapses, as well as the potency and therapeutic window of sFKN peptide, remain to be elucidated, our findings suggest that sFKN therapy could be beneficial for patients with NICS at least in the acute stages of the response to a noise insult and potentially for other patients with cochlear synaptopathy that affect understanding of speech or central auditory processing disorders.

### Limitations of the present study

The present study did not examine the optimal dosage, therapeutic window, or toxicology of soluble FKN or the duration and sexual dependence of observed synapse restoration with sFKN peptides. Additionally, the ability of sFKN in modulating macrophage density, morphological activation (transformative index), inflammatory profile, and phagocytic index was not undertaken in the present study. Before sFKN can be used clinically for noise-induced cochlear synaptopathy and hidden hearing loss, these studies are warranted.

## Data Availability

The original contributions presented in the study are included in the article/Supplementary material, further inquiries can be directed to the corresponding author.
