# Supplementary material for: Local delivery of soluble fractalkine (CX3CL1) peptide restores ribbon synapses after noise-induced cochlear synaptopathy
Source: Front Cell Neurosci. 2024 Oct 30;18:1486740. doi: 10.3389/fncel.2024.1486740 (PMC11557324; doi:10.3389/fncel.2024.1486740)
Supplement: Supplementary file 1 [file Data_Sheet_1.DOCX]

**List of Supplementary Materials**

Materials and Methods

**Real time-quantitative polymerase chain reaction (RT-qPCR).** The total RNA was extracted with Biorad-PureZOL (#7326880). Isolated RNA was treated with DNase 1, RNase-free (Thermo Scientific, #ENO521). The first strand cDNA was synthesized from 1 µg of total RNA using high-capacity RNA-to-cDNA kit (Applied Biosystems, #4388950). About 100ng/2µl of cDNA was used as a template for q-RT-PCR performed using TaqMan^TM^ Fast Advanced Master Mix (Applied Biosystems, #4444557) in ABI PRISM 7500 Fast. Expression level was analyzed using TaqMan® Gene expression assay kit for CX3CR1 and GAPDH (Applied Biosystems, # 4331182). The PCR program was as follows: UNG incubation at 50°C for 2 min, polymerase activation at 95°C for 10 min, Denaturation at 95°C for 15 sec, Anneal and extension at 60°C for 1min, total of 40 cycles were performed. Values were normalized to GAPDH and to the no noise exposed group and represented as fold change.

**Cochlear hair cell count.** Cochlear whole-mounts were processed for immunolabeling for hair cells. Both inner and outer hair cells were identified by their immunoreactivity for Myosin 7A. Hair cells were counted from the apical, middle, and basal region of the cochlea, as recorded in 20X objective images. Data was expressed as inner or outer hair cells in 300 µm of sensory epithelium (16).

**Supplementary figures and figure legends**

**
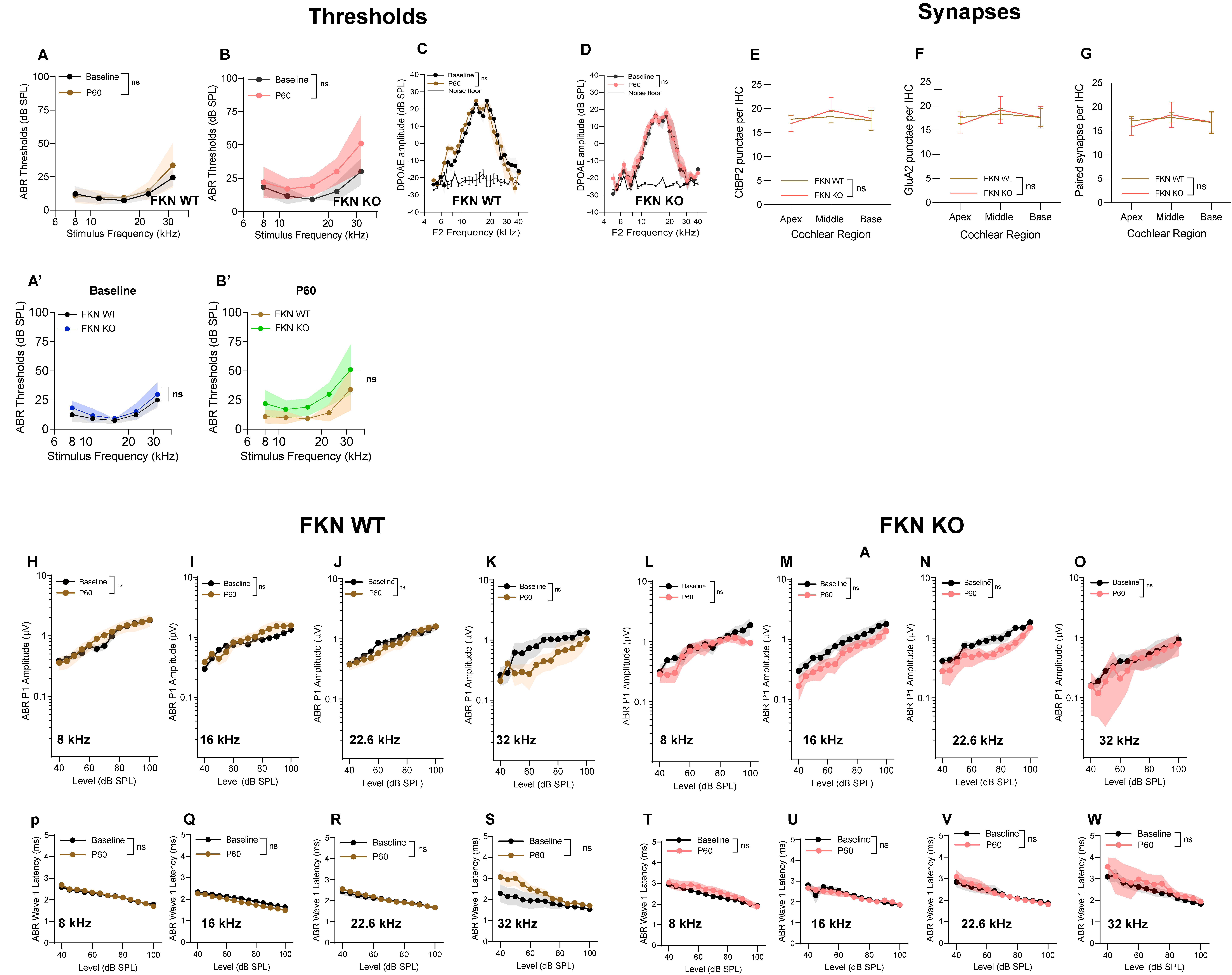
**

**Fig. S1.** **Gross hearing function in unexposed and uninjected FKN WT and FKN KO control mice.** (A & A’, B & B’) ABR thresholds at baseline (P45) and at experimental endpoint (P60) in FKN WT and FKN KO mice. 2-way ANOVA, Sidak’s multiple comparisons. DPOAE levels at baseline (P45) and at experimental endpoint (P60) in (C) FKN WT and (D) FKN KO mice. 2-way ANOVA, Sidak’s multiple comparisons. (E) CtBP2 puncta, (F) GluA2 puncta and (G) paired ribbon synapses per IHC in FKN WT and FKN KO at P60. 2-way ANOVA, Dunnett's multiple comparisons test. ABR peak I amplitude at baseline (P45) and at experimental endpoint (P60) in FKN WT mice at (H) 8 kHz, (I) 16 kHz, (J) 22.6 kHz and (K) 32 kHz. ABR peak I amplitude at baseline (P45) and at experimental endpoint (P60) in FKN KO mice at (L) 8 kHz, (M) 16 kHz, (N) 22.6 kHz and (O) 32 kHz. ABR peak I latency at baseline (P45) and at experimental endpoint (P60) in FKN WT mice at (P) 8 kHz, (Q) 16 kHz, (R) 22.6 kHz and (S) 32 kHz. ABR peak I latency at baseline (P45) and at experimental endpoint (P60) in FKN KO mice at (T) 8 kHz, (U) 16 kHz, (V) 22.6 kHz and (W) 32 kHz. 1-way ANOVA, Tukey’s multiple comparisons test (H-W). Values are mean ± SD. ns, non-significant comparison between baseline (P45) and at experimental endpoint (P60). N=6-7 mice per genotype.

**
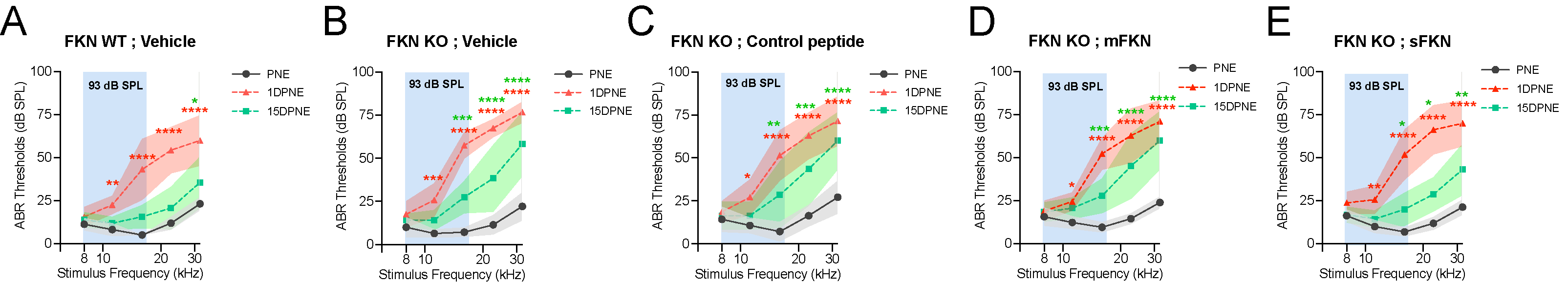
Fig. S2.** **ABR thresholds at pre-noise exposure (PNE), 1 DPNE and 15 DPNE.** ABR thresholds at pre-noise exposure, 1 day post noise exposure (DPNE) and 15 DPNE after 2 hours at 93 dB SPL at 8-16 kHz octave band noise (A) FKN WT mice treated with vehicle (N=8), and FKN KO mice treated with (B) vehicle (N=6) (C) control peptide (N=7) (D) membrane-bound FKN peptide (mFKN) (N=9) and (E) soluble FKN peptide (sFKN) (N=8). Values are means ± SD. *P < 0.05, **P < 0.01, ***P < 0.001, ****P < 0.0001 at respective stimulus frequency. *Represents comparison between PNE versus 1 DPNE (red asterisks) or 15 DPNE (green asterisks), 2-way ANOVA, Tukey’s multiple comparisons.

**
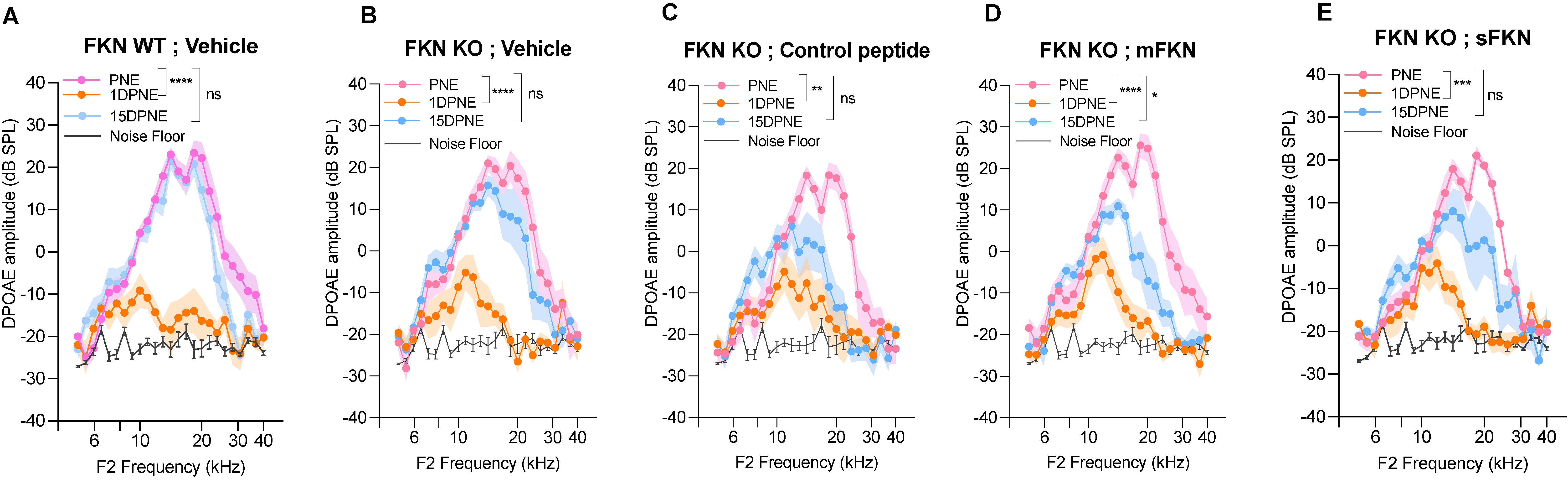
Fig. S3.** **DPOAE amplitude at pre-noise exposure (PNE), 1 DPNE and 15 DPNE.** DPOAE levels in (A) FKN WT mice treated with vehicle (N=8), and in FKN KO mice treated with (B) vehicle (N=6) (C) control peptide (N=7) (D) membrane-bound FKN peptide (mFKN) (N=10) and (E) soluble FKN peptide (sFKN) (N=8). Values are means ± SD. *P < 0.05, **P < 0.01, ***P < 0.001, ****P < 0.0001 and ns, non-significant at respective experimental time points. *Represents comparison between PNE and 1DPNE or 15 DPNE as shown with parenthesis, 2-way ANOVA, Dunnett's multiple comparisons test.

**
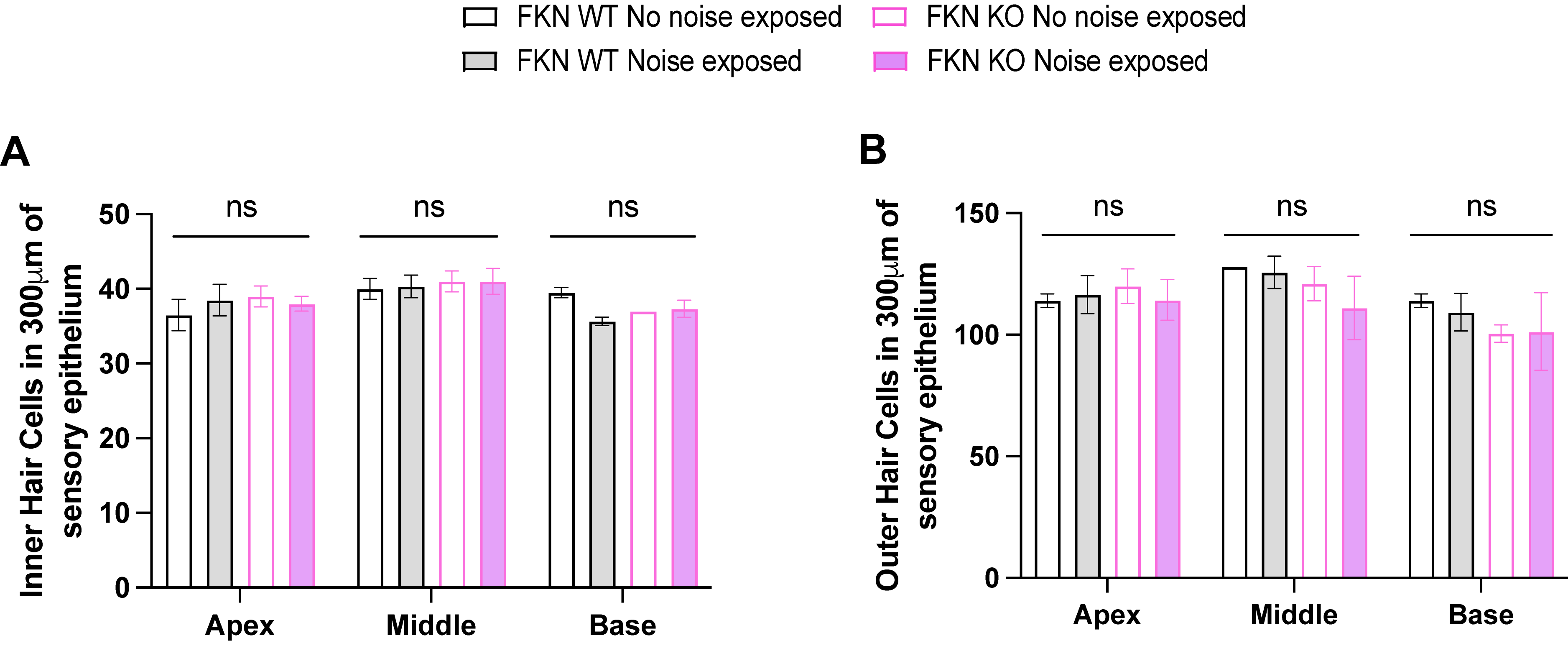
**

**Fig. S4.** **Hair cell density in FKN WT and FKN KO mice after NICS.** Density of cochlear (A) inner hair cells and (B) outer hair cells in no noise exposed (open bars) and noise exposed (filled bars) FKN WT and FKN KO mice shows no significant (ns) effect on hair cell survival between the genotypes. N=3 mice per genotype. 2-way ANOVA, Tukey’s multiple comparison test.


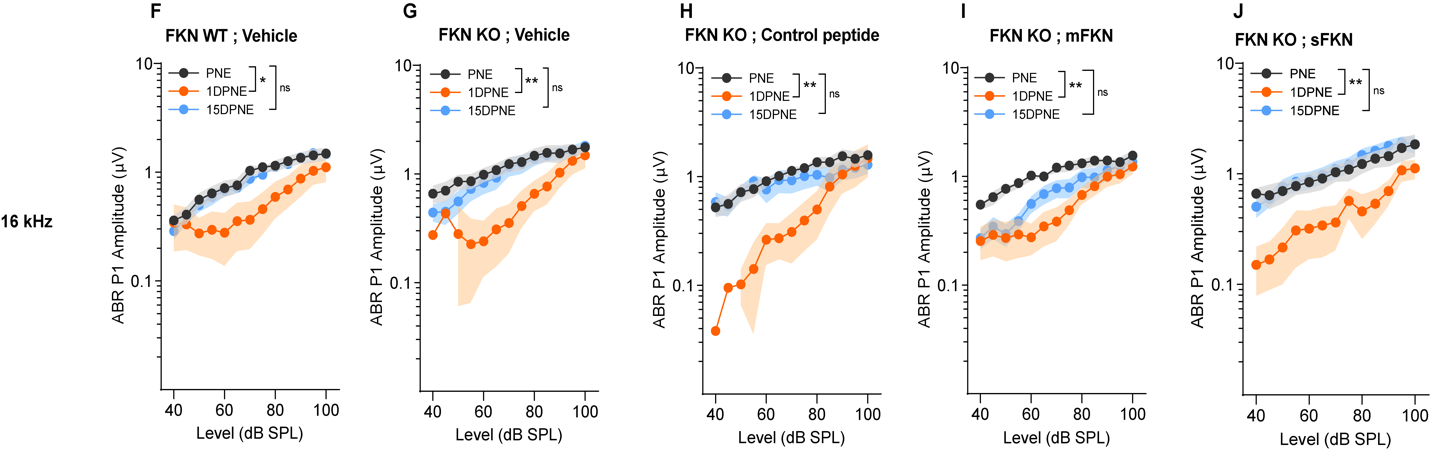


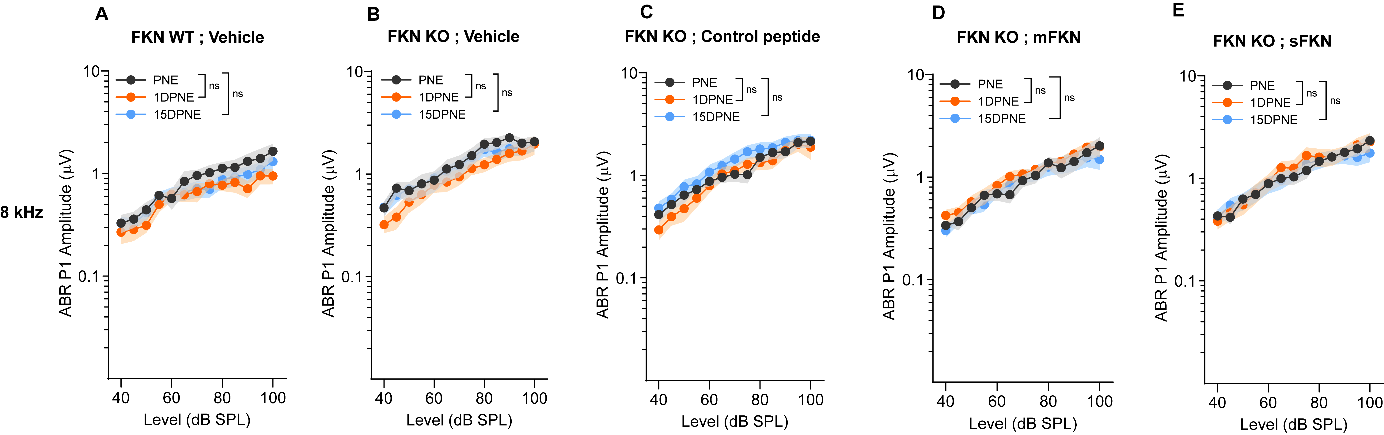


**
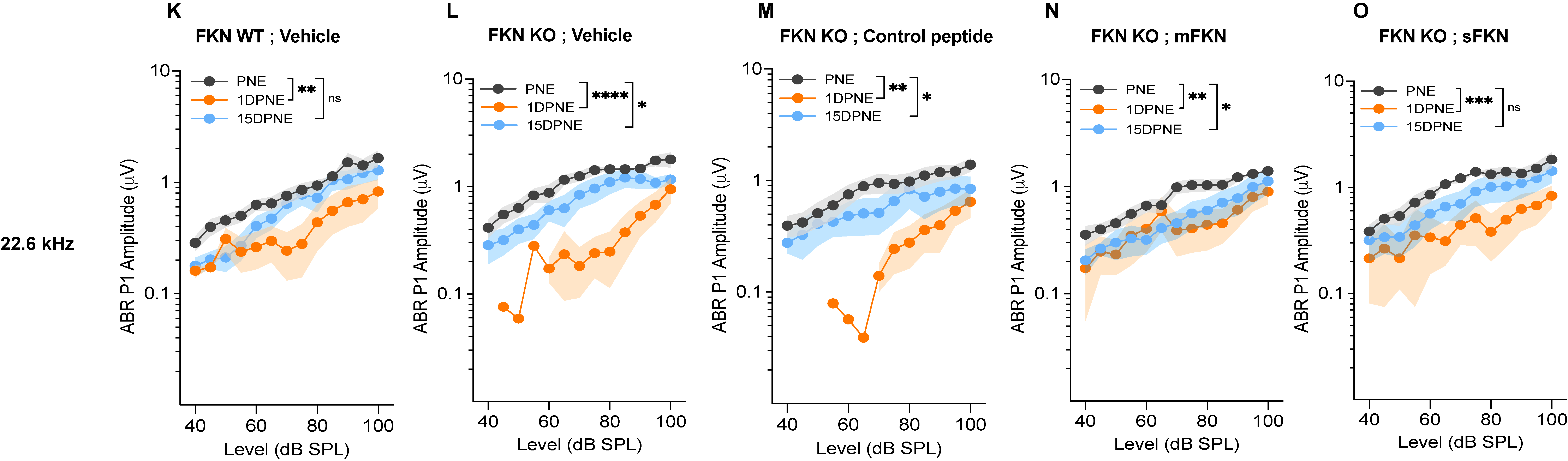
**

**Fig. S5.** **ABR Peak I amplitude at 8 kHz, 16 kHz and 22.6 kHz.** ABR Peak I amplitudes at 8 kHz (A) FKN WT mice treated with vehicle (N=8), FKN KO mice treated with (B) vehicle (N=6) (C) control peptide (N=7) (D) membrane-bound FKN peptide (mFKN) (N=10) and (E) soluble FKN peptide (sFKN) (N=8). At 16 kHz (F) FKN WT mice treated with vehicle (N=8), FKN KO mice treated with (G) vehicle (N=6) (H) control peptide (N=7) (I) membrane-bound FKN peptide (mFKN) (N=9) and (J) soluble FKN peptide (sFKN) (N=8). ABR Peak I amplitudes at 22.6 kHz (A) FKN WT mice treated with vehicle (N=8), FKN KO mice treated with (B) vehicle (N=6) (C) control peptide (N=7) (D) membrane-bound FKN peptide (mFKN) (N=10) and (E) soluble FKN peptide (sFKN) (N=8). Values are means ± SD. *P < 0.05, **P < 0.01, ***P < 0.001, **P < 0.0001 and ns, non-significant. *Represents the comparison between the experimental time points as indicated with parenthesis. 1-way ANOVA, Tukey’s multiple comparisons test.


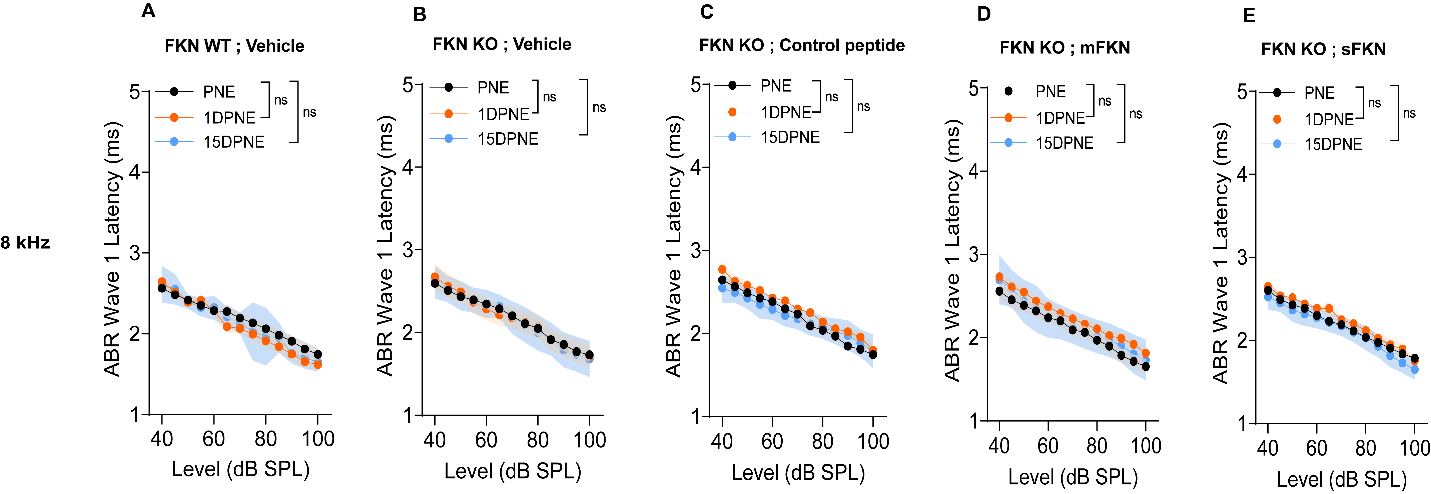


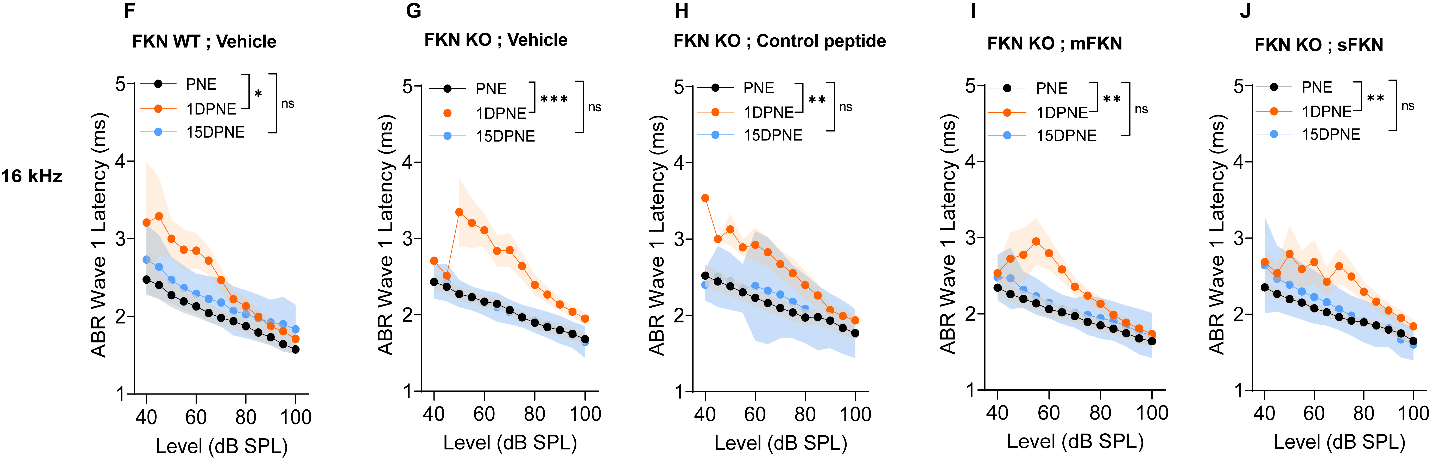


**
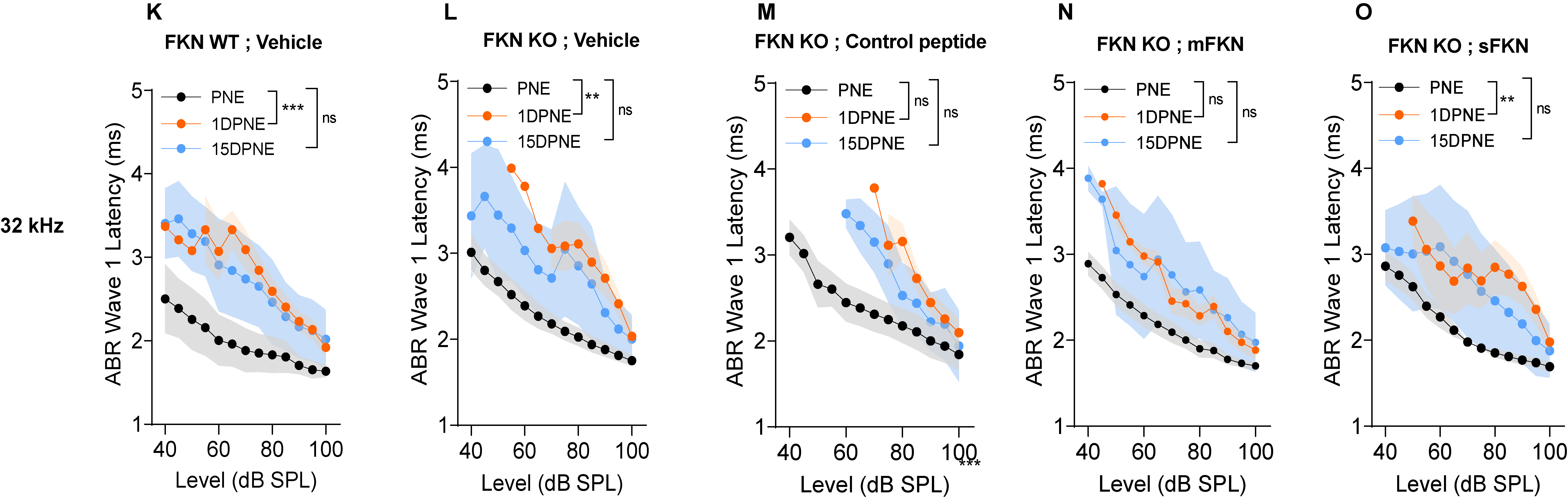
**

**Fig. S6. ABR Peak I latency at 8, 16, 32 kHz.** ABR Peak I latency at 8 kHz in (A) FKN WT mice treated with vehicle (N=8), and in FKN KO mice treated with (B) vehicle (N=6) (C) control peptide (N=7) (D) membrane-bound FKN peptide (mFKN) (N=10) and (E) soluble FKN peptide (sFKN) (N=8). At 16 kHz in (F) FKN WT mice treated with vehicle (N=8), and in FKN KO mice treated with (G) vehicle (N=6) (H) control peptide (N=7) (I) membrane-bound FKN peptide (mFKN) (N=9) and (J) soluble FKN peptide (sFKN) (N=8). At 32 kHz in (K) FKN WT mice treated with vehicle (N=8), and in FKN KO mice treated with (L) vehicle (N=6) (M) control peptide (N=7) (N) membrane-bound FKN peptide (mFKN) (N=10) and (O) soluble FKN peptide (sFKN) (N=8). Values are means ± SD. *P < 0.05, **P < 0.01, ***P < 0.001, and ns, non-significant. *Represents the comparison between the experimental time points as indicated with parenthesis. 1-way ANOVA, Tukey’s multiple comparisons test.

**
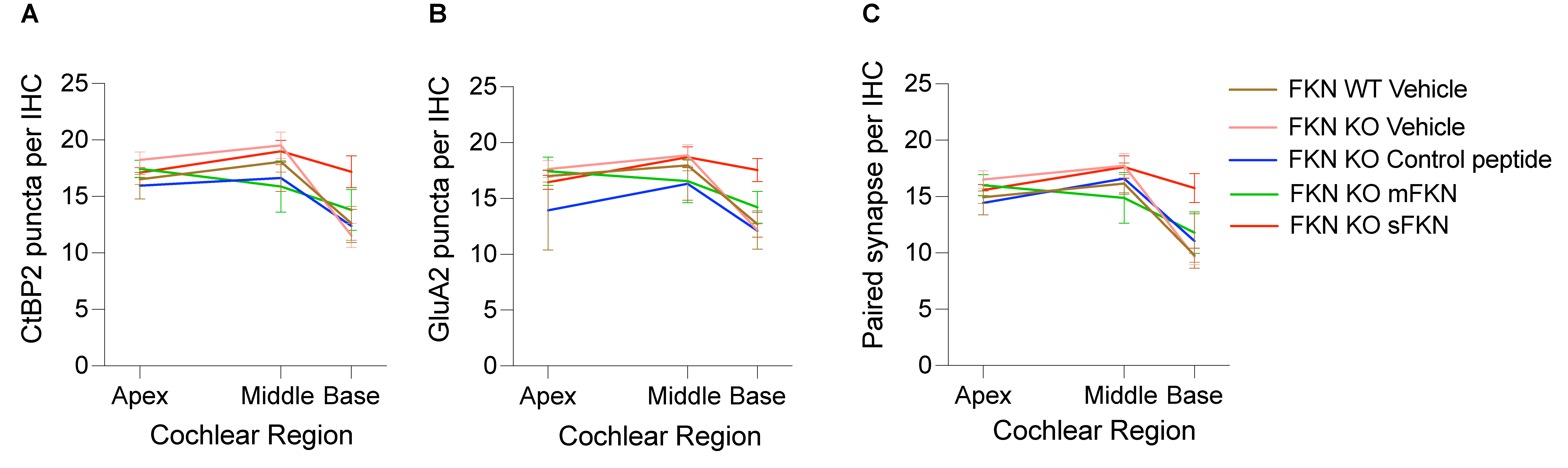
Fig. S7**. **Pre- and postsynaptic puncta and paired synapses per IHC along the cochlear length at 15 DPNE.** (A) CtBP2 puncta per IHC, (B) GluA2 puncta per IHC and (C) Paired synapse density per IHC at apex, middle and base cochlear regions. Values are mean ± SD. N= 5-9 mice per experimental group. Note, data shown in (A) does not represent ribbon “survival”.


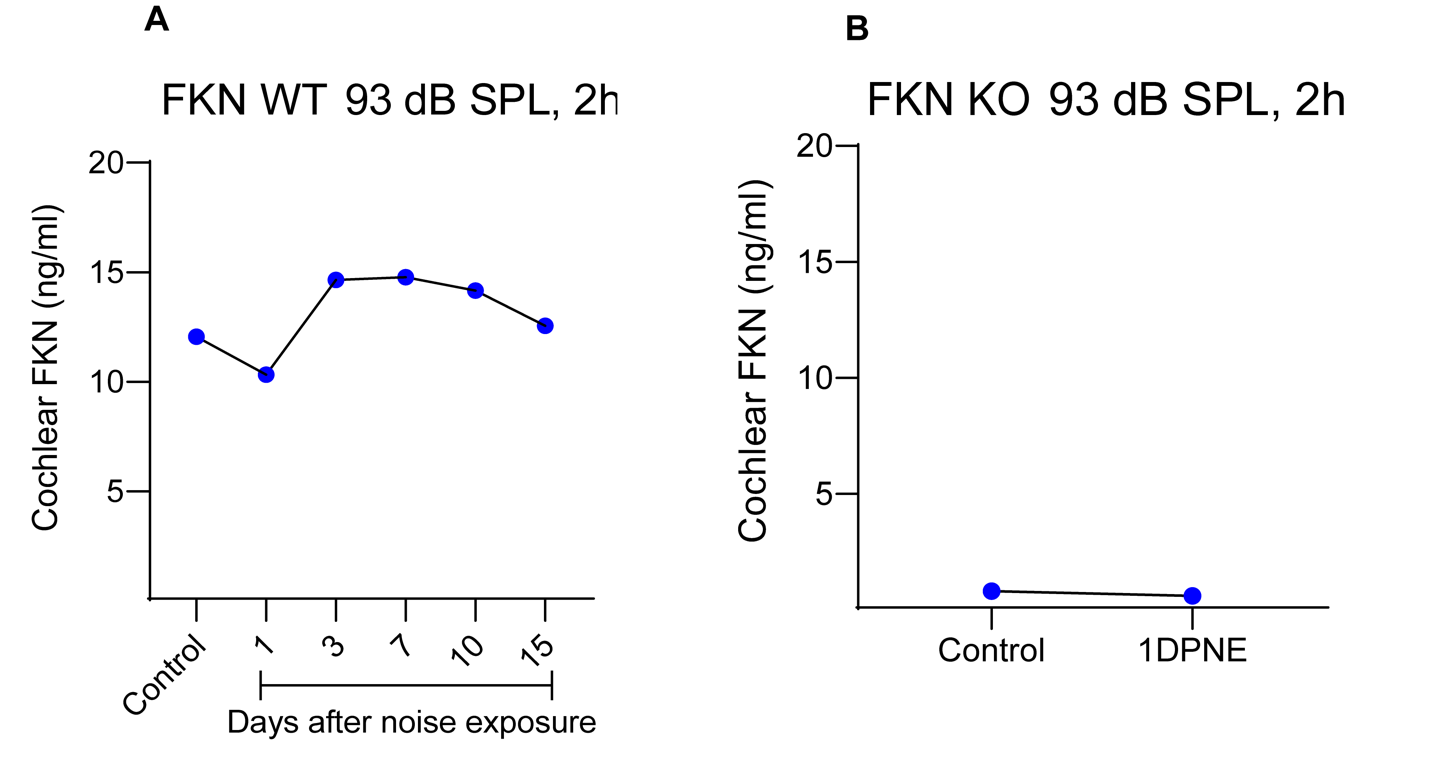
**Fig. S8. Cochlear endogenous soluble FKN levels after NICS.** ELISA based measurement of cochlear endogenous soluble FKN levels in (A) FKN WT mice and (B) FKN KO mice at different time points after NICS**.** Values are plotted as mean. N=6 cochleae from 3 different mice per time point per genotype. DPNE, days post noise exposure.

**
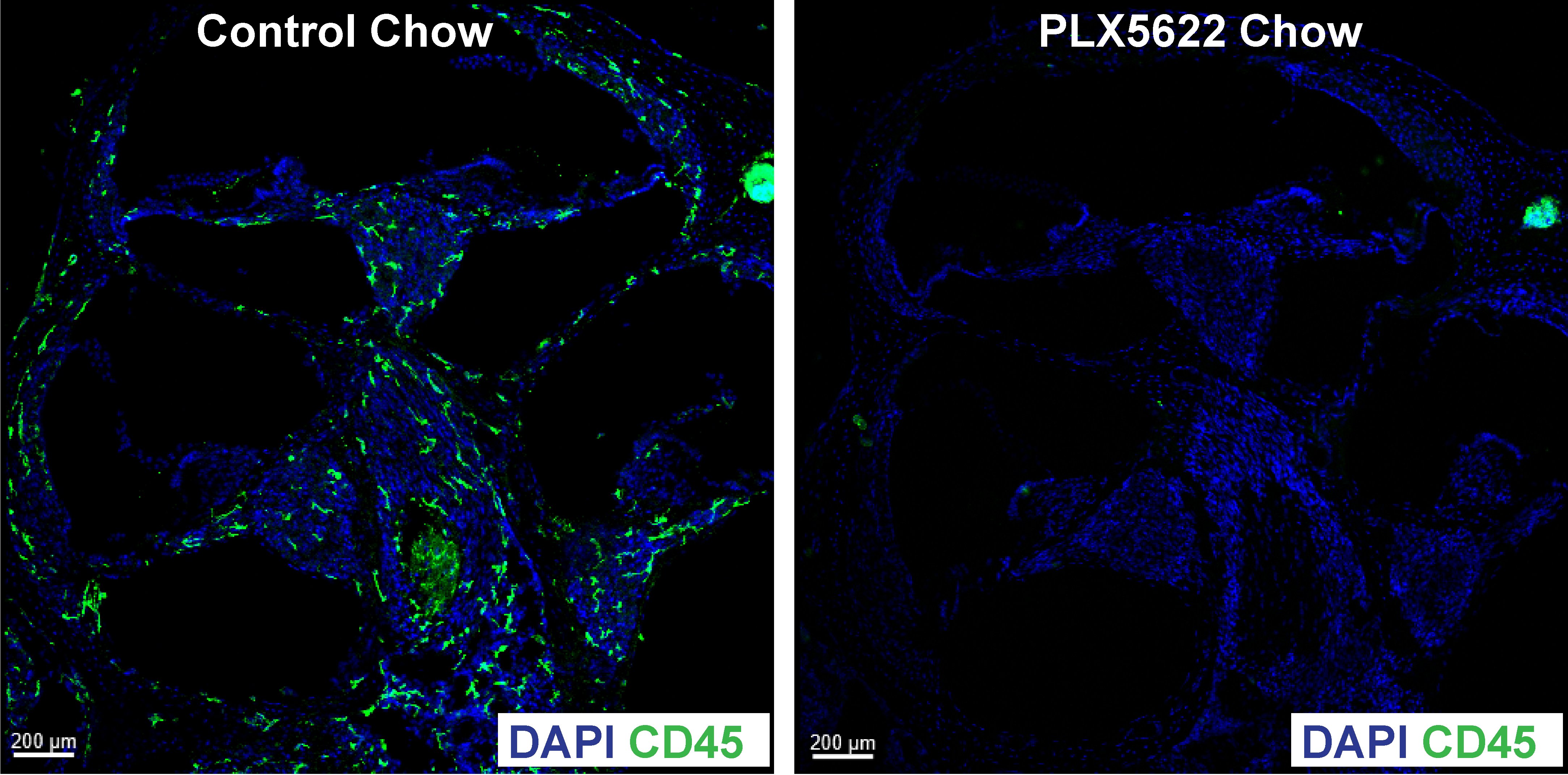
**

**Fig. S9. PLX5622-induced depletion of cochlear resident macrophages.** Representative images of cochlear mid-modiolar cross-sections from mature FKN WT mice fed on PLX5622 chow for 15 days show a robust depletion of cochlear resident macrophages (green, CD45 leukocytes) when compared to FKN WT mice fed on control chow without the PLX5622 compound for 15 days. Data is from same mice as shown in the main Fig. 5C-F. Also see reference Manickam et al., 2023 (13).


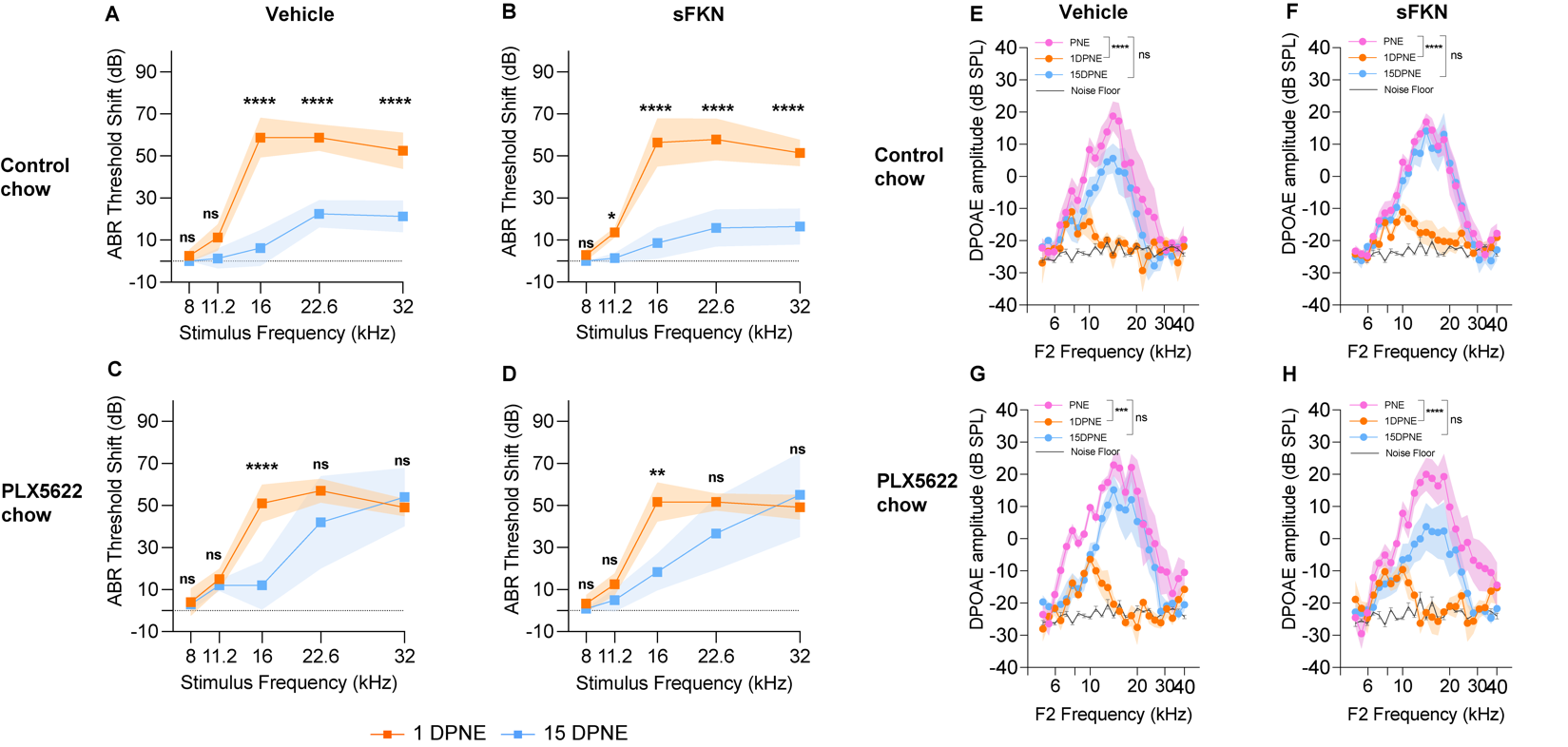
**Fig. S10. ABR thresholds shifts (A-D) and DPOEA levels (E-H) in vehicle- and sFKN-treated FKN WT mice in the presence (control chow) or absence (PLX5622 chow) of cochlear resident macrophages.** N=5-7 mice per experimental group. Values are means ± SD. *Represents comparison between 1DPNE and 15 DPNE (A-D). *Represents comparison between PNE and 1DPNE or 15 DPNE as shown with parenthesis (E-H). 2-way ANOVA, Sidak’s multiple comparisons. *P < 0.05, **P < 0.01, ***P < 0.001, ****P < 0.0001 and ns, non-significant. Dashed line represents threshold shifts prior to noise exposure (baseline, A-D).


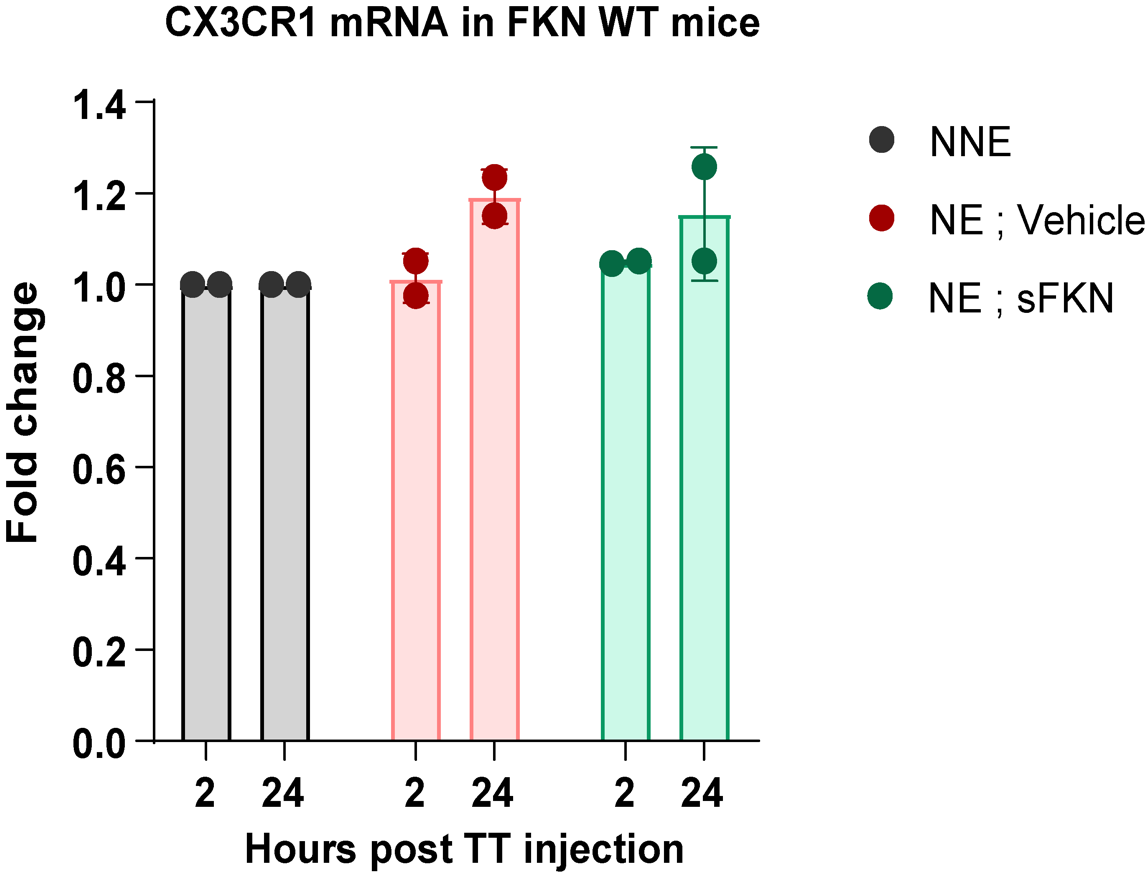


**Fig. S11**. **mRNA levels of cochlear CX_3_CR1 receptor.** CX_3_CR1 receptor mRNA measured by RT-qPCR in unexposed and noise exposed FKN WT mice after 2 and 24 hours of sFKN peptide TT injection. Values are mean ± SD. N=2 biological replicates per experimental group with 3 cochleae pooled from 3 different mice per group per biological replicate.


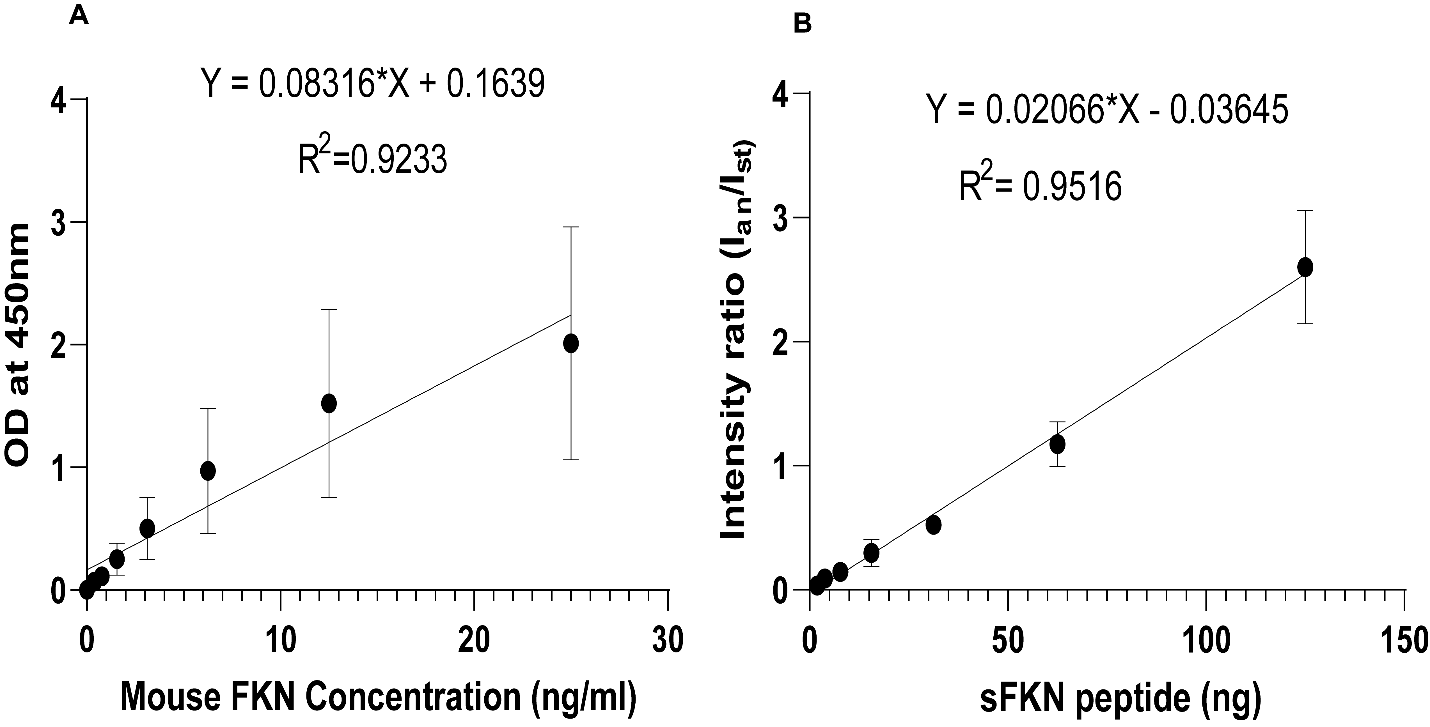


**Fig. S12.** **Standard curves for sFKN peptide for (A) ELISA and (B) MALDI-TOF-MS to detect the concentration of transtympanically injected sFKN peptide in the cochlea.**

**
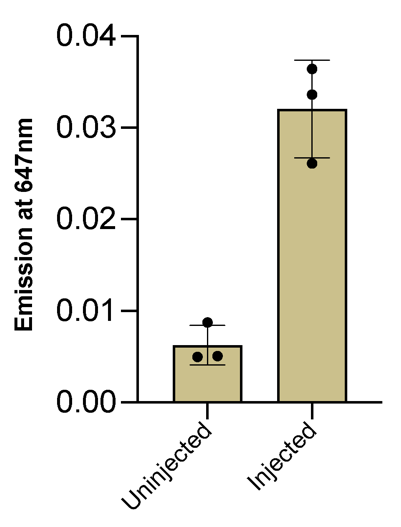
**

**Fig. S13. Mean fluorescence intensity of sFKN-Alexa Fluor 647 in cochlear perilymph at 3 hours after TT injection.** Values are mean ± SD. N=3 FKN WT mice per experimental group.
